# Synthesis and evaluation of MR probes for targeted-reporter imaging†
†Electronic supplementary information (ESI) available. See DOI: 10.1039/c7sc02217d



**DOI:** 10.1039/c7sc02217d

**Published:** 2017-06-13

**Authors:** Kirti Dhingra Verma, Justin O. Massing, Sarah G. Kamper, Christiane E. Carney, Keith W. MacRenaris, James P. Basilion, Thomas J. Meade

**Affiliations:** a Department of Biomedical Engineering , Case Center for Imaging Research , The NFCR Center for Molecular Imaging , Case Western Reserve University , Cleveland , Ohio 44106-7207 , USA; b Department of Chemistry , Molecular Biosciences, Neurobiology , Biomedical Engineering, and Radiology , Northwestern University , 2145 Sheridan Road , Evanston , Illinois 60208-3113 , USA

## Abstract

Visualizing disease heterogeneity remains a challenging task since most imaging agents are targeted to a single receptor. We describe the development of an MR platform able to report on multiple molecular events. Enzyme activation and enhanced cellular uptake of this modular probe make it suitable for subsequent targeted-reporter imaging applications.

## Abstract 2

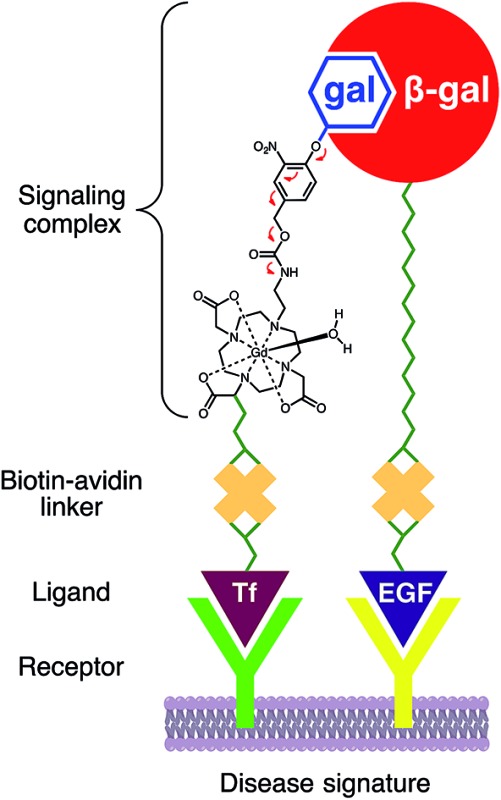
Visualizing disease heterogeneity remains a challenging task since most imaging agents are targeted to a single receptor.

## 

Extracellular receptors enable cells to interact with and adapt to their surrounding environment through a series of interwoven signalling cascades.^1^ Receptor expression levels therefore provide valuable insight with regard to cellular health. For instance, given the increased nutrient requirements necessary for uncontrolled cellular proliferation, cancerous cells are often characterized by the concerted overexpression of multiple cell surface receptors.^2^ Specifically, overexpression and amplification of epidermal growth factor receptor (EGFR) has been reported as the primary gain-of-function mutation observed in brain,^3,4^ breast,^5^ lung,^6^ ovarian,^7^ and skin cancers.^8^ Moreover, transferrin receptor (TfR) has been implicated in several cancers given the involvement of the iron-dependent enzyme ribonucleotide reductase during rapid DNA synthesis.^9,10^ Thus, targeted molecular imaging strategies able to report on the status of multiple biomarkers have the potential to function as innovative diagnostic agents during drug development.

The Basilion lab has demonstrated the ability to visualize multiple biomarkers using complementing β-galactosidase (β-gal) fragments targeted to EGFR and TfR.^11^ This approach relied on colocalization of complementing enzyme fragments at extracellular surfaces overexpressing the target receptors to restore enzyme activity, thus permitting their detection following colorimetric staining with X-gal *in vitro*. While this approach represents a first attempt at multimarker visualization, the colorimetric output is restricted to translucent specimens, thereby precluding longitudinal studies *in vivo*. More recently, Grimm and coworkers achieved *in vivo* imaging of multiple biomarkers by utilizing Cerenkov luminescence produced by targeted positron-emitting radionuclides to excite nearby bioactivatable fluorescent nanoparticles.^12^ However, the use of radionuclides make this imaging platform unsuitable for longitudinal studies given prolonged exposure to ionizing radiation. To overcome these limitations, we designed a complementary approach employing magnetic resonance (MR) imaging.

MR imaging has greatly impacted diagnostic medicine due to its high spatiotemporal resolution and unlimited penetration depth.^13,14^ To improve image contrast, paramagnetic agents are frequently administered to shorten the longitudinal (*T*_1_) or transverse (*T*_2_) relaxation times of nearby water protons. This increase in the observed water relaxation rate (1/*T*_*i*,obs_, *i* = 1, 2) yields a change in image contrast commensurate with the rate in the presence (1/*T*_*i*,p_) and absence (1/*T*_*i*,d_) of the paramagnetic species. With seven unpaired electrons and high magnetic moment, Gd(iii) chelates are the most commonly employed *T*_1_ agents. The efficiency with which these agents shorten *T*_1_ is termed relaxivity (*r*_1_), and is influenced by the number of bound waters (*q*), their residency lifetime (*τ*_m_), and the rotational correlation time of the complex (*τ*_R_). Modulating any one of the above variables upon interaction with a stimulus generates responsive MR probes.^15–23^

Here, we present a modular platform for the noninvasive visualization of enzyme activity and receptor expression. To achieve this aim we designed a Gd(iii) chelate that incorporates a proximal glycoside and a distal biotin that allow signal transduction and receptor targeting, respectively. The corresponding galactose residue is covalently tethered to the Gd(iii) core through a self-immolative linker capable of modulating the coordination chemistry about the metal center in a manner similar to that previously reported by our group.^24^ Biotinylation of the probe enables facile complexation with avidin, a tetrameric protein capable of binding four biotin molecules (*K*_d_ = 10^–15^ M).^25^ Complexation of three MR agents to one avidin was predicted to enable facile targeting to the desired receptor through subsequent complexation between the remaining subunit and the desired biotinylated ligand (*e.g.*, Tf). β-gal could then similarly be targeted to a separate receptor with the appropriate biotinylated ligand (*e.g.*, EGF). Overexpression of both receptors is therefore expected to result in colocalization, receptor-mediated internalization, and subsequent activation through targeted-reporter complex formation (Fig. 1).

**Fig. 1:**
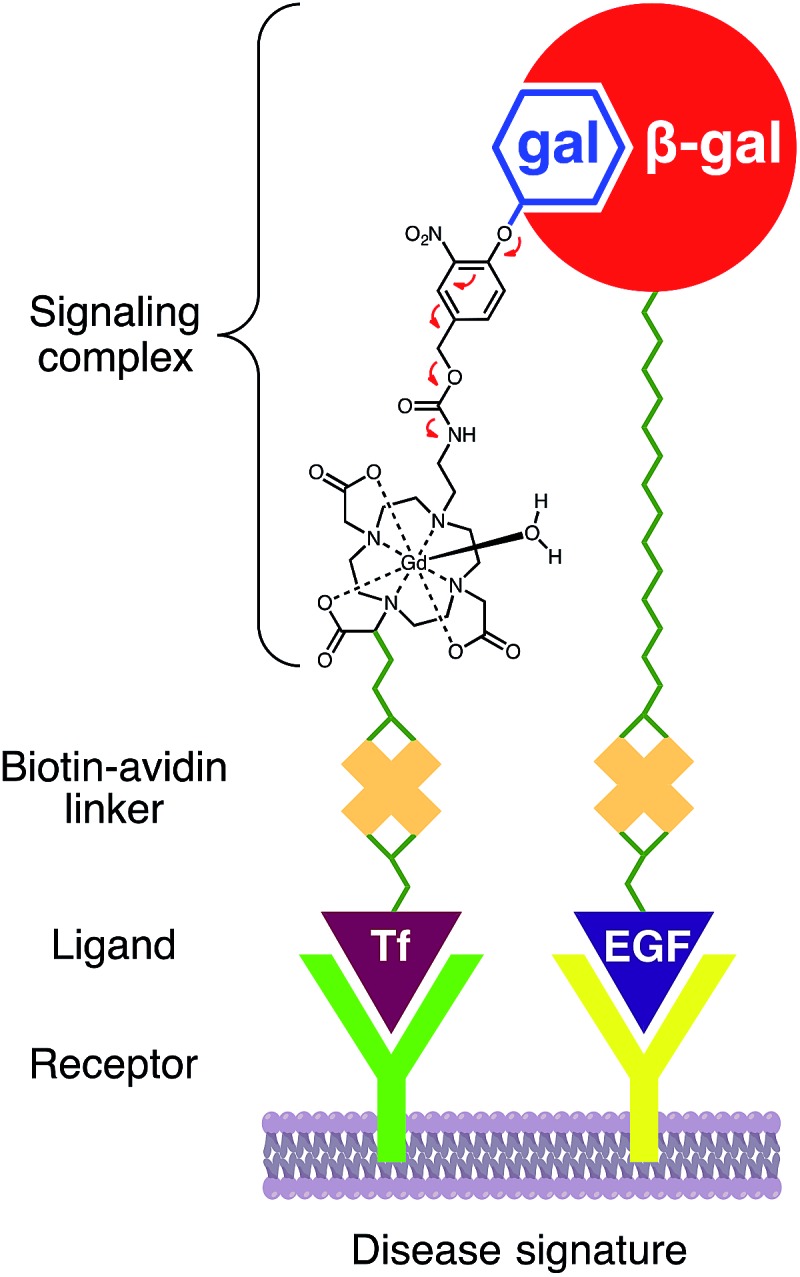
Formation of a targeted-reporter complex is achieved by conjugating a targeting ligand to an enzyme or its corresponding substrate. Thus, multiple cell surface receptors may be targeted simply by varying the ligands used. Cells overexpressing the receptors of interest enable positioning of the targeted enzyme and substrate close to one another, resulting in activation of the MR probe *via* a self-immolative cascade.

Briefly, 1,4,7,10-tetraazacyclododecane was protected at the 1 and 7 positions with Cbz-Cl^26^ prior to alkylation with ethyl bromoacetate; subsequent deprotection of the Cbz groups *via* catalytic hydrogenation yielded N-*trans* symmetrical material (Scheme S1†). Alkylation of this material with half an equivalent of 1-(*tert*-butyl)6-ethyl 2-bromohexanedioate^27^ (**3**) followed by acid-catalyzed cleavage of the *t*-butyl ester gave trisubstituted macrocycle in 34% over five steps. Reaction with **11**, followed by global deprotection and subsequent metalation yielded untargeted complex **Gd14** in 5% after preparative HPLC. Peptide coupling with norbiotinamine (**15**)^28^ and **16** ^29^ gave the desired biotinylated MR agents **Gd2** and **Gd1**, respectively (Table 1 and Scheme S3†). The corresponding Tb(iii) complexes were similarly generated to quantify *q* following luminescence lifetime decay measurements.

**Table 1:** Relaxivities and *q* measurements at 1.41 T and 37 °C in 100 mM MOPS, pH = 7.4

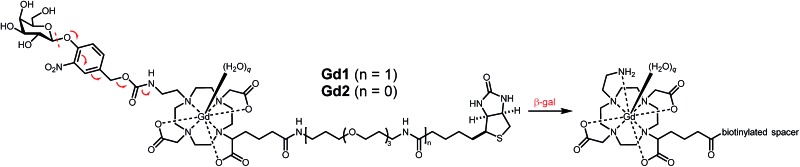				
	Relaxivity at 1.41 T (60 MHz)		*q*	
	(–) Avidin (mM^–1^ s^–1^)	(+) Avidin (mM^–1^ s^–1^)	(–) Avidin	(+) Avidin
**Gd1**	7.8	7.8	0.95	0.14
**Gd1** + β-gal	4.2	17.2	1.3	1
**Gd2**	8.6	19.3	1.0	0.9
**Gd2** + β-gal	4.2	n.a.	1.2	n.a.

**Gd1** and **Gd2** were assessed regarding their ability to influence the 1/*T*_1_ of nearby water protons at pH 7.4 in 100 mM MOPS buffer. *T*_1_ measurements made at 1.5 T and 37 °C yielded *r*_1_ values for **Gd1** and **Gd2** of 7.8 and 8.6 mM^–1^ s^–1^, respectively. Exposing each agent to β-gal afforded a measurable change in absorbance and 1/*T*_1_ (Fig. S19†). The apparent decrease in the latter is consistent with findings previously reported by our lab,^24^ and is likely due to the decrease in molecular weight upon enzyme activation.^30^ Both agents exhibited complete activation within three hours.

We next examined the ability of **Gd1** and **Gd2** to form host–guest complexes with avidin. This was confirmed by changes in 1/*T*_1_ and 1/*T*_2_ of nearby water protons upon successive additions of avidin. Measurements were performed identically (*vide supra*), and converted to *r*_1_ and *r*_2_ values following ICP-MS. As anticipated, **Gd2** exhibited an increase in both *r*_1_ (149%) and *r*_2_ (382%) upon complexation with avidin (Fig. S20†). This finding is expected given an increase in *τ*_R_ following complexation with biomacromolecules. Moreover, *r*_1_ and *r*_2_ values appeared to saturate upon binding of approximately three **Gd2** molecules to a single avidin tetramer, as has been seen before.^31^ While **Gd1** produced a similar increase in *r*_2_ (40%) that became saturated at the desired 4 : 1 stoichiometry, *r*_1_ remained largely unchanged throughout the experiment (Fig. 2). This response is important for subsequent targeted-reporter imaging and was later attributed to a concurrent decrease in *q* (*vide infra*). We therefore aimed to further establish **Gd1** as a viable candidate for such imaging applications by demonstrating its ability to drastically alter relaxation rates of nearby water protons upon activation while complexed with avidin.

**Fig. 2:**
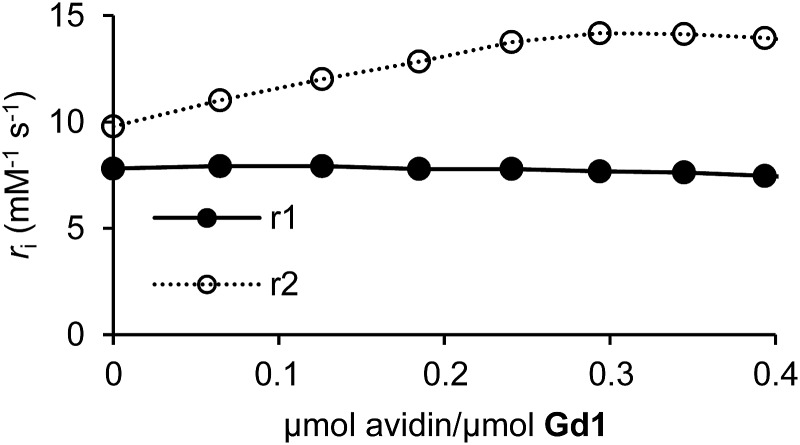
*r*_1_ and *r*_2_ as a function of avidin equivalents for **Gd1** (0.15 mM).

Attempts to activate **Gd2** complexed to avidin failed to alter *r*_1_, suggesting the proximity of avidin to the glycoside was sufficient to obviate β-gal recognition, and hence MR activation. However, **Gd1** readily underwent enzyme activation while complexed with avidin owing to the increased linker length. However, unlike before, activation of this macromolecular complex by β-gal afforded a desirable increase in *r*_1_ from 7.8 to 17.2 mM^–1^ s^–1^.

To understand these changes in *r*_1_ prior to, and following complexation with avidin and/or β-gal activation, luminescence lifetime measurements were performed with **Tb1** and **Tb2** (Table 1). As noted above, the constant *r*_1_ value for **Gd1** may be rationalized given a concurrent decrease in *q* and increase in *τ*_R_, the latter of which dominates at clinical field strengths.^13^ These findings indicate that avidin, like carbonate,^24,27,32^ is capable of efficiently masking the open coordination sites in **Gd1** to afford an MR agent exhibiting a desirable increase in *r*_1_ (130%) following activation by β-gal.

We determined the binding stoichiometry between this agent and avidin *via* a colorimetric assay using 2-(4-hydroxyphenylazo)benzoic acid (HABA). To a reconstituted HABA/avidin solution was titrated in a solution of **Gd1** and the change in HABA absorption at 500 nm monitored. Plotting the difference in absorption between each addition against the corresponding ratio of **Gd1** to avidin indicated the number of probes bound to each avidin (Fig. S21†). Upon the addition of four equivalents of **Gd1**, no observable change in the absorption of HABA was noted. These results confirm complete and efficient displacement of HABA from avidin in the presence of four equivalents of **Gd1**, suggesting negligible influence of the macrocyclic chelate on binding of the distal biotin to avidin.

Having established the desired 4 : 1 binding stoichiometry, we investigated the ability to target **Gd1** to cells overexpressing TfR. The corresponding **Gd1** : avidin : Tf (3 : 1 : 1) macromolecular construct was generated and cellular uptake subsequently examined in 9L cells known to overexpress TfR.^11^ Cells incubated with targeted agent (20 μM Gd(iii), 6.6 μM Tf) and untargeted agent (20 μM Gd(iii), 0 μM Tf) for 1, 2, 4, 8, or 24 hours show higher labelling for the targeted agent of approximately 0.3–0.4 fmol Gd(iii) per cell at all time points (Fig. 3). Additionally, at short incubation times of 1 and 2 hours, the magnitude of labelling by the untargeted agent is less than 0.1 fmol Gd(iii) per cell indicating that short incubation times are necessary to minimize the magnitude of non-specific binding. We therefore examined uptake in cells incubated with increasing concentrations of targeted and untargeted contrast agents for 2 hours (Fig. 3). At each concentration, there is significant preferential uptake of the targeted contrast agent. However, there is no significant increase in uptake of targeted contrast agent between incubations of 22 and 45 μM Gd(iii) indicating that cells have been saturated with the agent. These findings demonstrate that we are well within the limit of detection necessary for bioactivatable Gd(iii)-based agents (*vide infra*).^33–35^ However, attempts to visualize combinatorial biomarkers through colocalization of TfR-targeted **Gd1** and EGFR-targeted β-gal and subsequent activation of the former *in vitro* were unsuccessful. We conjectured that this finding was a result sluggish MR activation.

**Fig. 3:**
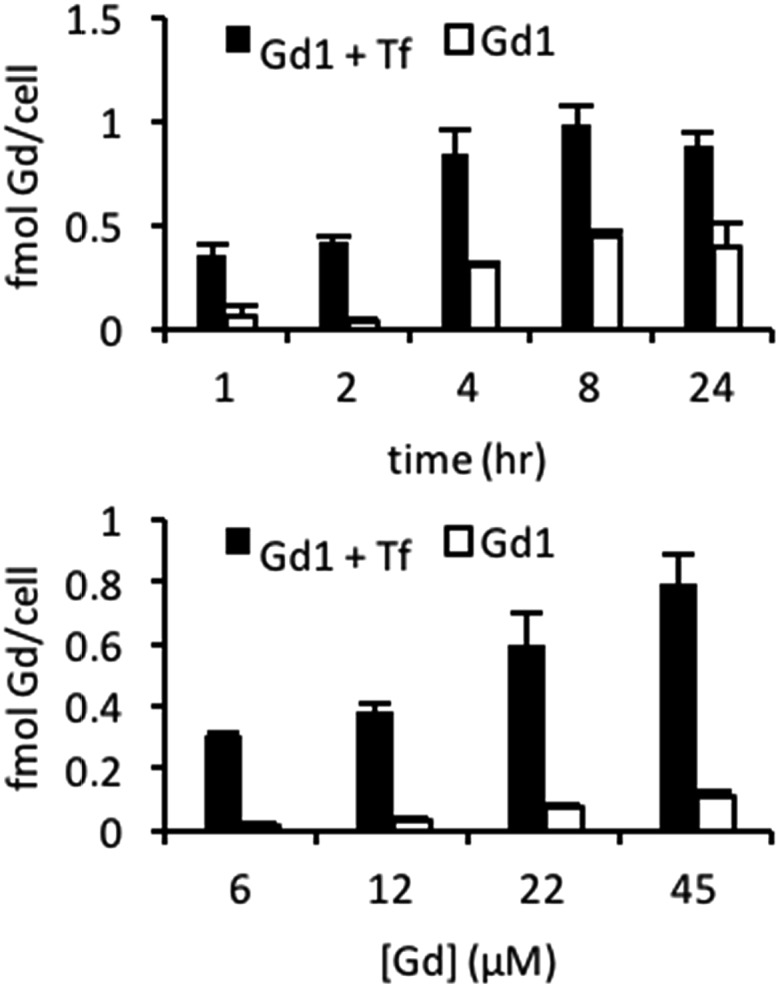
Time- (top) and concentration-dependent uptake (bottom) of TfR-targeted and untargeted **Gd1** illustrate significant uptake when complexed with biotinylated transferrin.

We investigated the kinetics associated with biotinylated β-gal activation required for subsequent targeted-reporter imaging. Biotinylated β-gal activity was established using *ortho*-nitrophenyl-β-galactoside (ONPG) through spectrophotometric measurements (Fig. S22†). Initial rates were determined and the reciprocal initial velocities (1/*V*_0_) plotted against the corresponding inverse concentrations (1/[ONPG]) to afford a Lineweaver–Burk plot (Fig. S23†). We examined ONPG activation by this enzyme while complexed with 0.25 equivalents of avidin (Fig. S24 and S25†). The corresponding host–guest interaction was found to exert little influence on the observed *k*_cat_, indicating this platform's suitability for subsequent targeted-reporter imaging applications.

Spectrophotometric measurements were repeated in the presence of **Gd1** (Fig. S26 and S27†). However, complexation of this agent with avidin (4 : 1) afforded negligible activation during the time course of the experiment, likely given the steric bulk of these complementing fragments. Despite this finding, we were able to visualize MR contrast enhancement following β-gal activation of **Gd1** complexed with avidin in solution (Fig. 4).

**Fig. 4:**
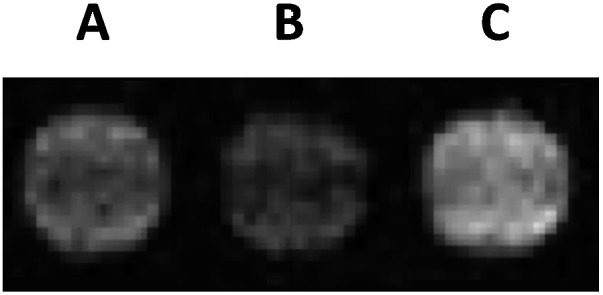
*T*_1_-weighted MR phantom images at 1.5 T. (A) **Gd1** (0.15 mM, 100 mM MOPS, pH = 7.4, 37 °C) alone, (B) **Gd1** complexed to avidin (20 U), and (C) **Gd1** complexed to avidin in the presence of β-gal (2.6 mU) after 4 h.

The detection limit of Gd(iii) *in vivo* depends on a variety of factors. These include the *r*_1_ of the agent, the intrinsic *T*_1_ values of the tissue being imaged, and the field strength of the magnet. The detection limit must therefore be determined for each imaging scenario. However, the limit at 9.4 T is commonly approximated to be 10 μM for a Gd(iii) chelate with an *r*_1_ of c. 7 mM^–1^ s^–1^.^34^ Whole proteome quantification of HeLa cells has shown that only proteins in the top 1% of expression level achieve cellular concentration of 10 μM.^35^ As a result, protein detection using Gd(iii) requires either a significant payload of Gd(iii) per binding event, or a target that amplifies the Gd(iii) uptake beyond simple stoichiometry. If a cell can be approximated to be 1 pL, a pellet of densely packed cells would require a total uptake value of 0.01 fmol Gd(iii)/cell. However, this value is unrealistic given the large degree of free space between cells. A more accurate approximation would be 0.1–1.0 fmol Gd(iii) per cell, thus providing a dynamic range for visualizing Gd(iii) *in vivo*. As stated above, although we are well within this range, the inability of targeted β-gal to activate targeted **Gd1** is due to either poor colocalization of these complementing fragments or inefficient activation under the conditions examined.

We have developed two bioresponsive MR agents for combinatorial imaging. **Gd1** and **Gd2** incorporate a self-immolative linker able to modulate agent efficiency in response to β-gal. While complexation between **Gd2** and avidin yielded an increase in *r*_1_ (124%), the resulting host–guest complex was incapable of undergoing enzyme activation. Complexation between **Gd1** and avidin failed to result in an *r*_1_ increase owing to a concurrent decrease in *q* and increase in *τ*_R_. However, this agent underwent a 130% increase in *r*_1_ following activation by β-gal while complexed with avidin. Moreover, **Gd1** displayed exceptional targeted cellular uptake necessary for combinatorial imaging. Future work is focused on further improving the change in *r*_1_ upon enzyme activation, and accelerating the kinetics associated with this process.
