# Supplementary material for: Synthesis and evaluation of MR probes for targeted-reporter imaging
Source: Chem Sci. 2017 Jun 13;8(8):5764–8. doi: 10.1039/c7sc02217d (PMC5621504; doi:10.1039/c7sc02217d)
Supplement: Supplementary file 1 [file SC-008-C7SC02217D-s001.pdf]

Electronic Supplementary Material (ESI) for Chemical Science.
This journal is © The Royal Society of Chemistry 2017

Supporting information

Synthesis and evaluation of MR probes for targeted-reporter imaging

Kirti Dhingra Verma,[1*] Justin O. Massing,[2*] Sarah G. Kamper,[2] Christiane E. Carney,[2] Keith W. MacRenaris,[2] James P. Basilion,[1#] and Thomas J. Meade[2#]

[1]Department of Biomedical Engineering, Case Center for Imaging Research, and the NFCR Center for Molecular Imaging, Case Western Reserve, Cleveland, Ohio 44106-7207

[2]Department of Chemistry, Molecular Biosciences, Neurobiology, Biomedical Engineering, and Radiology, Northwestern University, 2145 Sheridan Road, Evanston, Illinois 60208-3113, United States

[*]both authors have contributed equally to this study and share first authorship
[#]corresponding authors

1. **General methods** **S1**
2. **Synthetic schemes** **S2–S3**
3. **Experimental procedures** **S3–S12**
4. **General procedure for relaxation measurements** **S12**
5. **General procedures for photophysical studies** **S12–S13**
6. **MR imaging** **S14**
7. **General cell culture** **S14–S15**
8. **^{1}H and ^{13}C NMR spectra** **S16–S33**
9. **Enzyme activation measurements** **S34**
10. **Gd2 relaxivity measurements** **S34**
11. **Gd1:avidin binding stoichiometry** **S35**
12. **Enzyme kinetics** **S35–S38**
13. **Cell viability** **S39**

MATERIALS AND METHODS

General methods

All reactions were performed under an inert nitrogen atmosphere in oven-dried glassware equipped with a magnetic stir bar unless otherwise stated. $CHCl_3$, MeCN, DCM, and THF were purified by passage through a column of activated alumina,[1] while reagents were purified according to well-established procedures.[2] Product purification was carried out by flash chromatography using standard grade 60 Å 230–400 mesh silica gel (Macherey-Nagel). ALUGRAM Xtra SIL G 0.20 mm silica gel plates (Macherey-Nagel) were used for thin layer chromatography and visualized using UV light, ceric ammonium molybdate, or platinum stain. ^{1}H NMR spectra were recorded on Bruker Avance III 500 MHz spectrometer and are reported in

ppm relative to the deuterated solvent used. Spectral data are reported as s = singlet, d = doublet, t = triplet, q = quartet, p = pentet, hept = heptet, m = multiplet; coupling constant(s) in Hz; integration. Proton-decoupled ^{13}C NMR spectra were recorded on a Bruker Avance III 500 (125 MHz) and are reported in ppm relative to the deuterated solvent used. Reversed-phase semi-preparative HPLC was conducted on a Waters 19 x 250 mm^2 5 µm Atlantis T3 OBD column. The methods used on this system are as follows: (a) eluent A: 25 mM triethylammonium acetate (TEAA), pH 7.0, B: MeCN; gradient: 5% B to 50% B over 20 minutes; flow rate 15 mL/min, (b) eluent A: 25 mM TEAA, pH 7.0, B: MeCN; gradient: 5% B to 50% B over 25 minutes; flow rate 15 mL/min. Analytical HPLC was performed using a Waters 4.6 x 250 mm^2 5 µm Atlantis T3 column using the above methods with a flow rate of 1 mL/min. **3**,[3] **15**,[4] and **16**[5] were synthesized as previously reported.

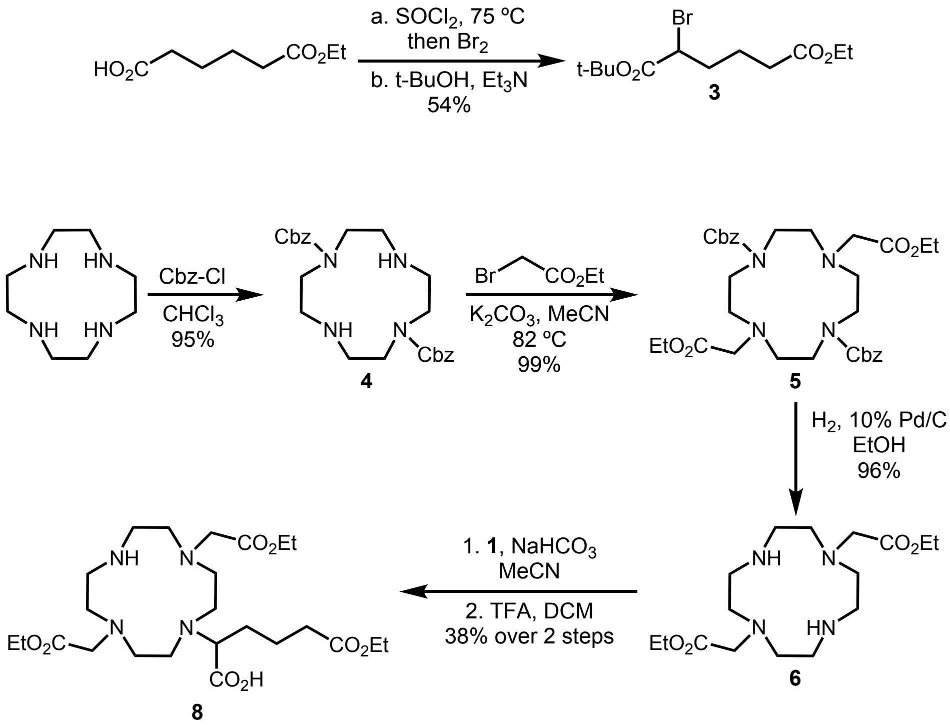

Scheme S1. Synthesis of the trisubstituted macrocyclic core **8**.

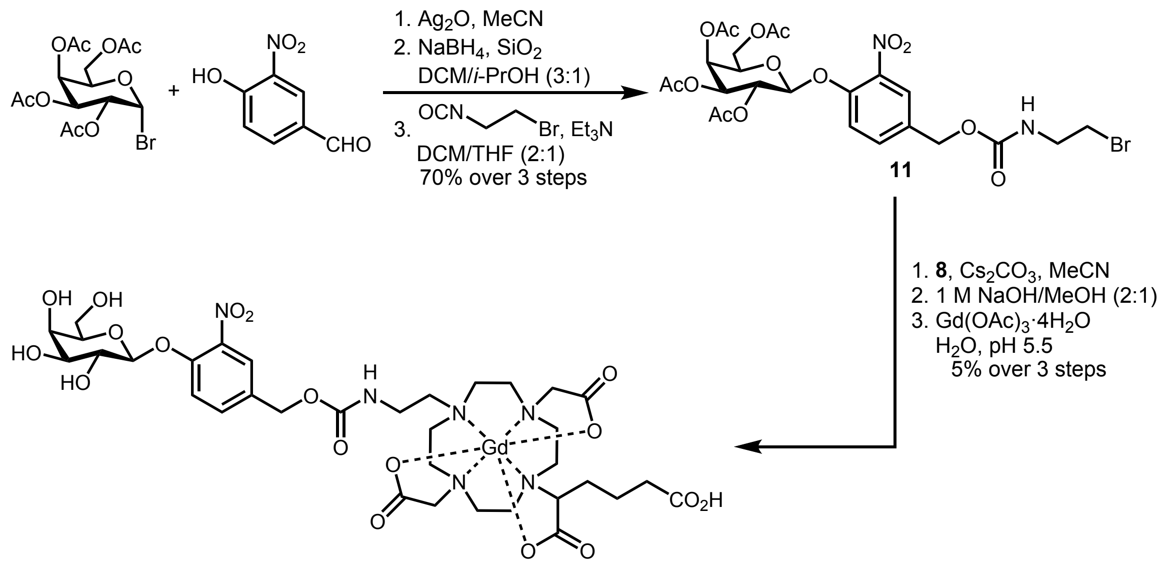

Gd14

Scheme S2. Synthesis of MR probe **Gd14** responsive toward β-gal with a free carboxylic acid for subsequent peptide coupling.

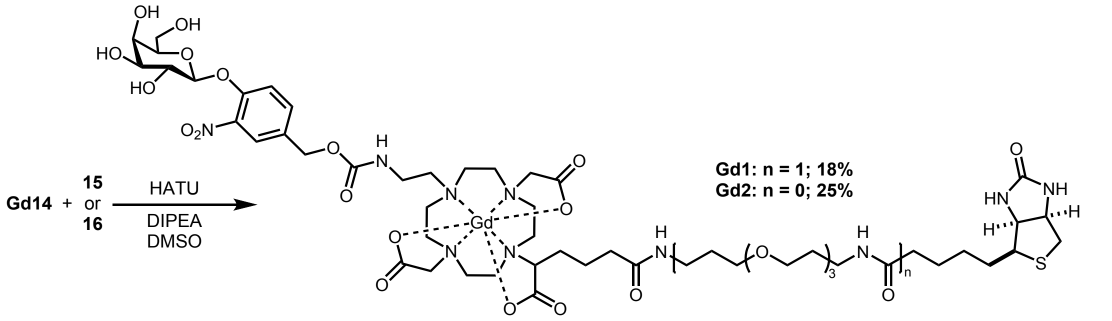

Scheme S3. Peptide coupling to afford the target biotinylated MR agent responsive toward β-gal.

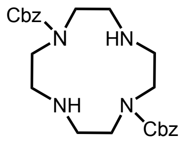

dibenzyl 1,4,7,10-tetraazacyclododecane-1,7-dicarboxylate (4). To a solution of cyclen (2.796 g, 16.23 mmol) in $CHCl_3$ (176 mL) at 0 ºC was added Cbz-Cl (4.6 mL, 32 mmol) dropwise. The reaction mixture was allowed to slowly warm to room temperature with continued stirring for 14 h before concentrating under reduced pressure. The resulting white residue was suspended in Et_2O (170 mL) and isolated by vacuum filtration.

Isolated material was dissolved in 3 M NaOH (150 mL) and extracted with $CHCl_3$ (6 x 30 mL). Combined organic fractions were dried over Na_2SO_4 and dried *in vacuo* to yield **4** as a viscous yellow oil (6.686 g, 95%). ^{1}H NMR (500 MHz, $CDCl_3$) δ 7.40 – 7.27 (m, 10H), 5.15 (s, 4H), 3.42 (dq, *J* = 13.8, 5.0 Hz, 8H), 2.84 (ddt, *J* = 55.7, 39.1, 4.9 Hz, 8H); ^{13}C NMR (126 MHz, $CDCl_3$) δ 156.95, 156.91, 136.91, 136.83, 128.65, 128.62, 128.12, 128.06, 128.01, 127.92, 67.26, 67.18, 51.34, 50.97, 50.90, 50.46, 49.80, 49.32, 48.70, 48.39; LC/MS (ESI): Exact mass calcd. for $C_{24}H_{32}N_4O_4$ $[M+H]^+$, 441.250. Found 441.283.

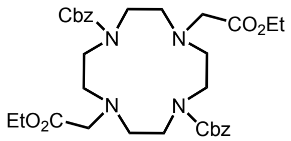

dibenzyl 4,10-bis(2-ethoxy-2-oxoethyl)-1,4,7,10-tetraazacyclododecane-1,7-dicarboxylate (5). **4** (5.823 g, 13.22 mmol) and K_2CO_3 (4.593 g, 33.23 mmol) were combined in MeCN (74 mL) and the resulting suspension brought to reflux. After 1 h, ethyl bromoacetate (2.9 mL, 26 mmol) was added dropwise over 10 min to the refluxing suspension. Continued refluxing for 18 h. The reaction mixture was then vacuum filtered through Celite and the resulting filtrate concentrated under reduced pressure. The corresponding residue was dissolved in DCM (50 mL) and washed with H_2O (2 x 10 mL) and brine (50 mL). The organic layer was dried over Na_2SO_4, concentrated *in vacuo*, and purified by flash chromatography on silica (95.5/4/0.5, DCM/MeOH/NH_4OH, R_f = 0.33) to afford the title compound as a pale yellow oil (8.276 g, 99%). ^{1}H NMR (500 MHz, $CDCl_3$) δ 7.35 – 7.24 (m, 10H), 5.11 (s, 4H), 4.10 (d, *J* = 9.4 Hz, 4H), 3.42 (s, 12H), 2.99 – 2.70 (m, 8H), 1.23 (t, *J* = 7.3 Hz, 6H); ^{13}C NMR (126 MHz, $CDCl_3$) δ 171.25, 156.53, 136.91, 128.54, 128.03, 127.97, 67.09, 60.37, 55.29, 54.56, 47.14, 46.67, 14.36; LC/MS (ESI): Exact mass calcd. for $C_{16}H_{32}N_4O_4$ $[M+H]^+$, 613.324. Found 613.22.

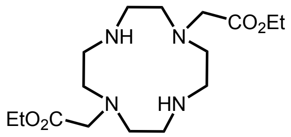

diethyl 2,2'-(1,4,7,10-tetraazacyclododecane-1,7-diyl)diacetate (6). **5** (7.317 g, 11.95 mmol) and 10% Pd/C (0.738 g) were combined in EtOH (220 mL) and the reaction mixture purged with N_2, then H_2. Maintained a H_2 atmosphere

for 15 h. The suspension was then vacuum filtered through Celite and the filtrate concentrated to afford the title compound (3.951 g, 96%). ^{1}H NMR (500 MHz, $CDCl_3$) δ 4.14 (q, *J* = 7.1 Hz, 4H), 3.41 (s, 4H), 2.79 (s, 8H), 2.60 (t, *J* = 5.1 Hz, 8H), 1.25 (t, *J* = 7.1 Hz, 6H); ^{13}C NMR (126 MHz, $CDCl_3$) δ 171.70, 60.46, 56.38, 52.20, 45.74, 14.42; LC/MS (ESI): Exact mass calcd. for $C_{16}H_{32}N_4O_4$ $[M+H]^+$, 345.250. Found 345.346.

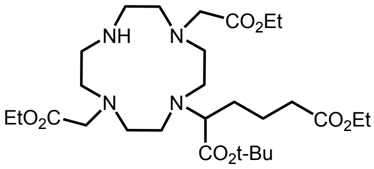

1-(*tert*-butyl) 6-ethyl 2-(4,10-bis(2-ethoxy-2-oxoethyl)-1,4,7,10-tetraazacyclododecan-1-yl)hexanedioate (7). To a stirring suspension of **6** (2.656 g, 7.711 mmol) and $NaHCO_3$ (0.659 g, 7.84 mmol) in MeCN (80 mL) was added dropwise a solution of **3** (1.192 g, 3.855 mmol) in MeCN (60 mL). The reaction mixture was filtered through Celite after 20 h. Concentration under reduced pressure followed by flash chromatography on silica (90/9/1, MeCN/H_2O/sat'd KNO_3, R_f = 0.65) afforded the title compound as an amber oil (1.597 g, 72%). ^{1}H NMR (500 MHz, $CDCl_3$) δ 4.14 (dq, *J* = 16.8, 7.1 Hz, 6H), 3.49 (q, *J* = 17.8 Hz, 4H), 3.31 (dd, *J* = 8.0, 4.4 Hz, 1H), 3.16 (ddd, *J* = 32.1, 9.8, 4.2 Hz, 7H), 2.98 (ddd, *J* = 13.6, 8.3, 4.9 Hz, 2H), 2.92 – 2.81 (m, 4H), 2.50 (dt, *J* = 14.1, 3.4 Hz, 2H), 2.41 (dq, *J* = 6.7, 2.9 Hz, 2H), 1.80 – 1.57 (m, 4H), 1.46 (s, 9H), 1.26 (dt, *J* = 8.6, 7.1 Hz, 11H); ^{13}C NMR (126 MHz, $CDCl_3$) δ 173.49, 171.48, 171.07, 82.12, 62.01, 60.87, 60.51, 55.55, 51.31, 50.70, 49.67, 46.27, 33.82, 29.23, 28.40, 22.28, 14.38; LC/MS (ESI): Exact mass calcd. for $C_{28}H_{52}N_4O_8$ $[M+H]^+$, 573.386. Found 573.437.

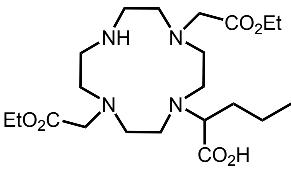

2-(4,10-bis(2-ethoxy-2-oxoethyl)-1,4,7,10-tetraazacyclododecan-1-yl)-6-ethoxy-6-oxohexanoic acid (8). **7** (1.6 g, 2.8 mmol) was dissolved in 95/2.5/2.5, v/v/v, TFA/H_2O/TIPS (22.5 mL) and the reaction sealed for 7 h. The reaction mixture was then concentrated and purified by flash chromatography on silica (90/0/1, MeCN/H_2O/sat'd KNO_3, R_f = 0.15) to afford the title compound as a clear colorless oil (0.72 g, 50%) ^{1}H NMR (500 MHz,

$CDCl_3$) δ 4.19 – 3.99 (m, 6H), 3.57 – 3.29 (m, 8H), 3.06 (dt, *J* = 26.2, 12.2 Hz, 8H), 2.88 (d, *J* = 10.9 Hz, 4H), 2.72 (s, 2H), 2.32 (tq, *J* = 16.4, 9.2, 8.1 Hz, 2H), 1.92 – 1.72 (m, 2H), 1.58 (qt, *J* = 10.4, 6.2, 5.7 Hz, 2H), 1.21 (q, *J* = 7.3 Hz, 9H); ^{13}C NMR (126 MHz, $CDCl_3$) δ 173.15, 172.53, 171.01, 63.40, 60.88, 60.43, 54.53, 49.47, 43.49, 33.88, 23.69, 22.71, 14.27; LC/MS (ESI): Exact mass calcd. for $C_{24}H_{44}N_4O_8$ $[M+H]^+$, 517.324. Found 517.351.

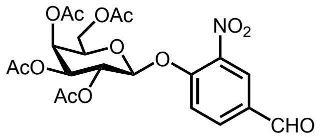

(2*R*,3*S*,4*S*,5*R*,6*S*)-2-(acetoxymethyl)-6-(4-formyl-2-nitrophenoxy)tetrahydro-2*H*-pyran-3,4,5-triyl triacetate (9). To a solution of (2R,3S,4S,5R,6R)-2-(acetoxymethyl)-6-bromotetrahydro-2H-pyran-3,4,5-triyl triacetate (1.776 g, 4.320 mmol) and 4-hydroxy-3-nitrobenzaldehyde (1.219 g, 7.294 mmol) in MeCN (40 mL) was added Ag_2O (4.420 g, 19.07 mmol). The resulting yellow suspension was protected from light and stirred for 18 h before being filtered through Celite. The filtrate was concentrated under reduced pressure, dissolved in EtOAc (130 mL), and washed with saturated $NaHCO_3$ (60 mL, then 7 x 20 mL). The organic layer was dried over Na_2SO_4 and concentrated under reduced atmosphere as a pale yellow solid (2.247 g, 99%). ^1H NMR (500 MHz, $CDCl_3$) δ 9.98 (s, 1H), 8.30 (d, *J* = 2.1 Hz, 1H), 8.07 (dd, *J* = 8.6, 2.1 Hz, 1H), 7.48 (d, *J* = 8.6 Hz, 1H), 5.59 (dd, *J* = 10.4, 7.9 Hz, 1H), 5.49 (dd, *J* = 3.5, 1.2 Hz, 1H), 5.21 (d, *J* = 7.9 Hz, 1H), 5.13 (dd, *J* = 10.5, 3.4 Hz, 1H), 4.26 (dd, *J* = 11.1, 6.8 Hz, 1H), 4.22 – 4.08 (m, 3H), 2.19 (s, 3H), 2.13 (s, 3H), 2.08 (s, 3H), 2.02 (s, 3H); ^{13}C NMR (126 MHz, $CDCl_3$) δ 188.67, 170.39, 170.22, 169.31, 153.59, 141.38, 134.09, 131.61, 127.00, 118.94, 100.23, 71.96, 70.49, 67.73, 66.71, 61.50, 20.81, 20.77, 20.74, 20.70; LC/MS (ESI): Exact mass calcd. for $C_{21}H_{23}NO_{13}$ $[M+HCO_2]^-$, 542.115. Found 542.075.

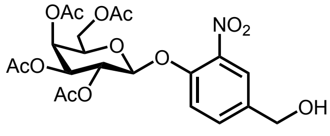

(2*R*,3*S*,4*S*,5*R*,6*S*)-2-(acetoxymethyl)-6-(4-(hydroxymethyl)-2-nitrophenoxy)tetrahydro-2*H*-pyran-3,4,5-triyl triacetate (10). **7** (1.835 g, 3.689 mmol) and SiO_2 (3.321 g) were suspended in 3/1, DCM/*i*-PrOH (40 mL) and

cooled to 0 °C. Addition of $NaBH_4$ (0.308 g, 8.14 mmol) afforded a bright yellow suspension with slight effervescence. After 4 h, the reaction mixture was quenched with ice-cold H_2O (30 mL) and filtered through Celite. The isolated organic layer was then washed with brine (30 mL), dried over Na_2SO_4, and concentrated *in vacuo* as an off-white solid (1.531 g, 83%). ^{1}H NMR (500 MHz, $CDCl_3$) δ 7.80 (d, J = 2.2 Hz, 1H), 7.51 (dd, J = 8.6, 2.2 Hz, 1H), 7.34 (d, J = 8.5 Hz, 1H), 5.53 (dd, J = 10.5, 7.9 Hz, 1H), 5.46 (dd, J = 3.5, 1.2 Hz, 1H), 5.10 (dd, J = 10.5, 3.4 Hz, 1H), 5.05 (d, J = 7.9 Hz, 1H), 4.72 (d, J = 5.0 Hz, 2H), 4.25 (dd, J = 11.3, 7.0 Hz, 1H), 4.16 (dd, J = 11.4, 6.2 Hz, 1H), 4.09 – 4.03 (m, 1H), 2.18 (s, 3H), 2.12 (s, 3H), 2.07 (s, 3H), 2.01 (s, 3H); ^{13}C NMR (126 MHz, $CDCl_3$) δ 170.47, 170.33, 170.28, 169.60, 148.57, 141.50, 137.28, 131.86, 123.36, 120.17, 101.01, 71.54, 70.69, 68.00, 66.86, 63.59, 61.47, 20.80, 20.78, 20.71; LC/MS (ESI): Exact mass calcd. for $C_{21}H_{25}NO_{13}$ $[M+Na]^+$, 522.122. Found 522.024.

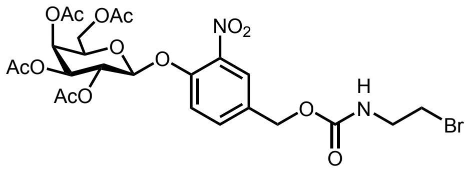

(2*R*,3*S*,4*S*,5*R*,6*S*)-2-(acetoxymethyl)-6-(4-((((2-bromoethyl)carbamoyl)oxy)methyl)-2-nitrophenoxy)tetrahydro-2*H*-pyran-3,4,5-triyl triacetate (11). To a colorless solution of **10** (1.518 g, 3.040 mmol) in 2/1, THF/DCM (135 mL) was added Et_3N (1.7 mL, 12 mmol), affording a bright yellow color. To this was added 2-bromoethyl isocyanate (0.587 g, 3.91 mmol), and the reaction mixture stirred for 12 h before being concentrated under reduced pressure and purified by flash chromatography on silica (40% hexanes in EtOAc, R_f = 0.38) to afford the title compound as a white foam (1.678 g, 85%). ^{1}H NMR (500 MHz, $CDCl_3$) δ 7.80 (d, J = 2.2 Hz, 1H), 7.51 (dd, J = 8.7, 2.2 Hz, 1H), 7.34 (d, J = 8.6 Hz, 1H), 5.53 (dd, J = 10.5, 7.9 Hz, 1H), 5.46 (dd, J = 3.4, 1.2 Hz, 1H), 5.24 (d, J = 6.7 Hz, 1H), 5.15 – 5.02 (m, 4H), 4.24 (dd, J = 11.4, 6.9 Hz, 1H), 4.16 (dd, J = 11.4, 6.1 Hz, 1H), 4.10 – 4.04 (m, 1H), 3.60 (q, J = 5.9 Hz, 2H), 3.47 (t, J = 5.8 Hz, 2H), 2.18 (s, 3H), 2.12 (s, 3H), 2.06 (s, 3H), 2.00 (s, 3H); ^{13}C NMR (126 MHz, $CDCl_3$) δ 170.41, 170.27, 170.23, 169.49, 155.78, 149.13, 141.37, 133.29, 132.75, 124.78, 119.93, 100.86, 71.58, 70.64, 67.93, 66.82, 65.13, 61.45, 42.90,

32.39, 20.80, 20.77, 20.69; LC/MS (ESI): Exact mass calcd. for $C_{24}H_{29}BrN_2O_{14}$ [M+Na]$^+$, 671.070. Found 670.986.

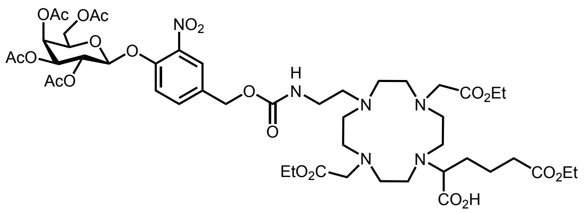

2-(4,10-bis(2-ethoxy-2-oxoethyl)-7-(2-((((3-nitro-4-(((2*S*,3*R*,4*S*,5*S*,6*R*)-3,4,5-triacetoxy-6-(acetoxymethyl)tetrahydro-2*H*-pyran-2-yl)oxy)benzyl)oxy)carbonyl)amino)ethyl)-1,4,7,10-tetraazacyclododecan-1-yl)-6-ethoxy-6-oxohexanoic acid (12). **8** (0.114 g, 0.221 mmol), **11** (0.175 g, 0.269 mmol), and Cs_2CO_3 (0.097 g, 0.30 mmol) were combined in MeCN (10 mL). The suspension was stirred for 72 h then filtered through Celite and concentrated under reduced pressure. Flash chromatography on silica (90/0/1, MeCN/H_2O/sat'd KNO_3, R_f = 0.66) afforded the title compound as a clear colorless oil (0.124 g, 52%). ^1H NMR (500 MHz, $CDCl_3$) δ 7.80 – 7.70 (m, 1H), 7.52 (t, *J* = 9.0 Hz, 1H), 7.38 – 7.29 (m, 1H), 5.54 – 5.47 (m, 1H), 5.44 (d, *J* = 3.4 Hz, 1H), 5.15 – 4.99 (m, 5H), 4.35 – 3.95 (m, 8H), 3.66 – 3.29 (m, 11H), 3.17 (s, 1H), 3.09 – 2.91 (m, 3H), 2.83 – 2.55 (m, 6H), 2.38 – 2.26 (m, 1H), 2.15 (d, *J* = 1.6 Hz, 3H), 2.08 (d, *J* = 2.7 Hz, 3H), 2.04 (d, *J* = 2.6 Hz, 3H), 1.98 (s, 4H), 1.82 – 1.46 (m, 2H), 1.33 – 1.10 (m, 7H); ^{13}C NMR (126 MHz, $CDCl_3$) δ 173.65, 173.51, 172.89, 172.16, 170.95, 170.71, 170.44, 170.41, 170.39, 170.23, 170.15, 170.13, 169.44, 156.83, 156.32, 149.02, 148.95, 141.11, 140.94, 133.34, 133.26, 133.01, 132.86, 124.68, 124.38, 119.76, 119.57, 100.62, 71.51, 71.47, 70.62, 70.60, 67.93, 66.89, 66.85, 64.82, 64.73, 63.88, 63.71, 62.80, 61.42, 61.37, 61.23, 60.83, 60.63, 55.44, 55.31, 52.05, 51.63, 50.92, 50.20, 49.97, 49.08, 48.83, 45.71, 40.21, 35.61, 33.63, 33.55, 22.35, 22.29, 20.75, 20.71, 20.64, 14.29, 14.27, 14.22; LC/MS (ESI): Exact mass calcd. for $C_{48}H_{73}N_6O_{22}$ [M+H]$^+$, 1085.478. Found 1085.600.

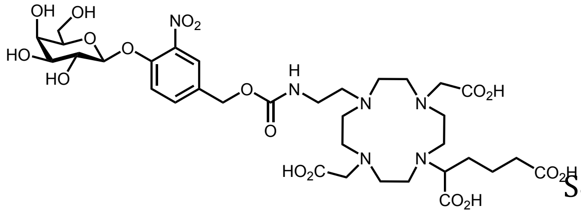

2-(4,10-bis(carboxymethyl)-7-(2-((((3-nitro-4-(((2S,3R,4S,5R,6R)-3,4,5-trihydroxy-6-

(hydroxymethyl)tetrahydro-2H-pyran-2-yl)oxy)benzyl)oxy)carbonyl)amino)ethyl)-1,4,7,10-tetraazacyclododecan-1-yl)hexanedioic acid (13). **12** (0.885 g, 0.816 mmol) was dissolved in MeOH (20 mL) and allowed to chill in an ice bath. Meanwhile, a 0.1 M NaOH solution was prepared and similarly placed in the ice bath. Once sufficiently chilled, 0.1 M NaOH (39 mL) was carefully added to the ice-cold solution of **12** and the reaction progress monitored by LC/MS. After 2 h, MeOH was removed by rotary evaporation and the resulting aqueous solution carefully neutralized with 1 M HCl before being lyophilized. The resulting material was then purified by preparative HPLC using method A. Fractions were combined and lyophilized to afford the title compound as a clear colorless oil (0.075 g, 11%).

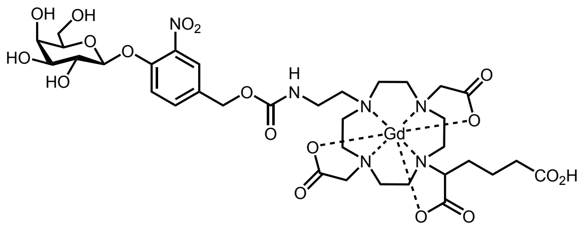

2-(4,10-bis(carboxymethyl)-7-(2-((((3-nitro-4-(((2S,3R,4S,5R,6R)-3,4,5-trihydroxy-6-(hydroxymethyl)tetrahydro-2H-pyran-2-yl)oxy)benzyl)oxy)carbonyl)amino)ethyl)-1,4,7,10-tetraazacyclododecan-1-yl)hexanedioic acid, gadolinium(III) (Gd14). **13** (0.131 g, 0.157 mmol) was dissolved in H_2O (15 mL) and the pH adjusted to between 9 and 10 with 1 M NaOH. $Gd(OAc)_3 \cdot 4H_2O$ (0.096 g, 0.236 mmol) was then added, the reaction mixture adjusted to pH 5.5, and its progress monitored by LC/MS. Following complete complexation, the pH of the reaction mixture was adjusted to between 9 and 10 and $Gd(OH)_3$ removed by centrifugation. The pH of the resulting solution was adjusted to 7 and subsequently filtered through a 0.20 μm filter. The corresponding filtrate was lyophilized to afford the title compound as a waxy solid (0.155 g, 99%) LC/MS (ESI): Exact mass calcd. for $C_{34}H_{50}GdN_6O_{18}$ $[M+H]^+$, 988.242. Found 988.219.

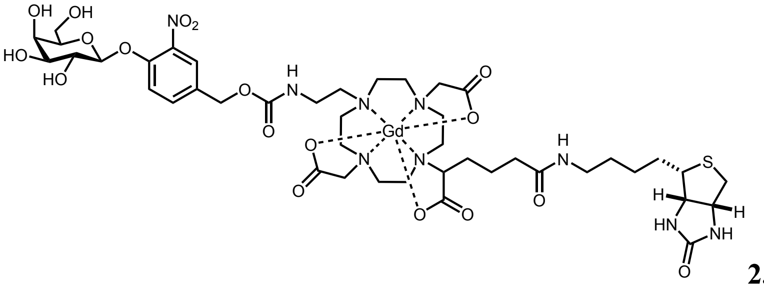

2,2'-(4-(1-carboxy-5-oxo-5-((5-((3aS,4S,6aR)-2-oxohexahydro-1H-thieno[3,4-d]imidazol-4-yl)pentyl)amino)pentyl)-10-(2-((((3-nitro-4-(((2S,3R,4S,5R,6R)-3,4,5-trihydroxy-6-(hydroxymethyl)tetrahydro-2H-pyran-2-yl)oxy)benzyl)oxy)carbonyl)amino)ethyl)-1,4,7,10-tetraazacyclododecane-1,7-diyl)diacetic acid, gadolinium(III) (Gd2). **Gd14** (0.030 g, 0.03 mmol), DIPEA (0.015 mL, 0.086 mmol), and HATU (0.017 g, 0.045 mmol) were combined in DMSO (1 mL) and allowed to stir for 10 min under inert atmosphere. To this was added dropwise a solution of **15** (0.015 g, 0.044 mmol) and DIPEA (0.015 mL, 0.086 mmol) in DMSO (0.8 mL). The resulting reaction mixture was allowed to stir for an additional 24 h before being purified by preparative HPLC (method B) as a white solid (0.009 g, 25%). LC/MS (ESI): Exact mass calcd. for $C_{43}H_{64}GdN_9O_{18}S$ $[M-H]^-$, 1183.325. Found 1181.23.

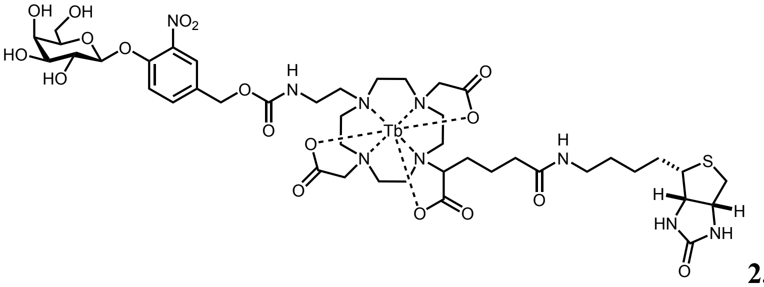

2,2'-(4-(1-carboxy-5-oxo-5-((5-((3aS,4S,6aR)-2-oxohexahydro-1H-thieno[3,4-d]imidazol-4-yl)pentyl)amino)pentyl)-10-(2-((((3-nitro-4-(((2S,3R,4S,5R,6R)-3,4,5-trihydroxy-6-(hydroxymethyl)tetrahydro-2H-pyran-2-yl)oxy)benzyl)oxy)carbonyl)amino)ethyl)-1,4,7,10-tetraazacyclododecane-1,7-diyl)diacetic acid, terbium(III) (Tb2). The title compound was synthesized and purified in an analogous

fashion to that reported for **Gd2**. LC/MS (ESI): Exact mass calcd. for $C_{43}H_{64}TbN_9O_{18}S$ $[M-H]^-$, 1184.33. Found 1184.10.

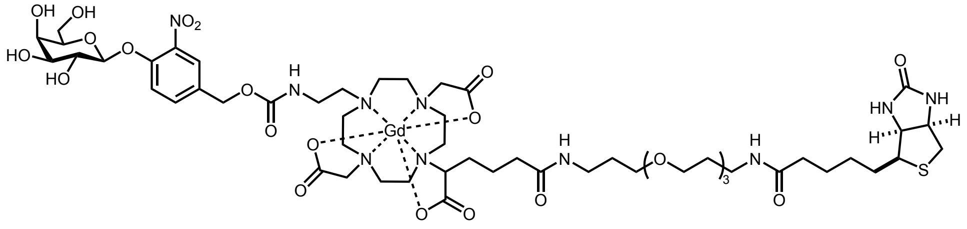

2,2'-(4-(1-carboxy-5,21-dioxo-25-(2-oxohexahydro-1*H*-thieno[3,4-*d*]imidazol-4-yl)-10,13,16-trioxa-6,20-diazapentacosyl)-10-(2-((((3-nitro-4-(((2*S*,3*R*,4*S*,5*R*,6*R*)-3,4,5-trihydroxy-6-(hydroxymethyl)tetrahydro-2*H*-pyran-2-yl)oxy)benzyl)oxy)carbonyl)amino)ethyl)-1,4,7,10-tetraazacyclododecane-1,7-diyl)diacetic acid, gadolinium(III) (Gal-Gd-PEG-biotin). **Gd14** (0.030 g, 0.030 mmol), DIPEA (0.015 mL, 0.086 mmol), and HATU (0.017 g, 0.045 mmol) were combined in DMSO (1 mL) and allowed to stir for 10 min under inert atmosphere. To this was added dropwise a solution of **16** (0.025 g, 0.045 mmol) and DIPEA (0.015 mL, 0.086 mmol) in DMSO (0.8 mL). The resulting reaction mixture was allowed to stir for an additional 24 h before being purified by preparative HPLC (method B) as a white solid (0.008 g, 19%). LC/MS (ESI): Exact mass calcd. for $C_{54}H_{85}GdN_{10}O_{22}S$ $[M+Na]^+$, 1438.470. Found 1438.513.

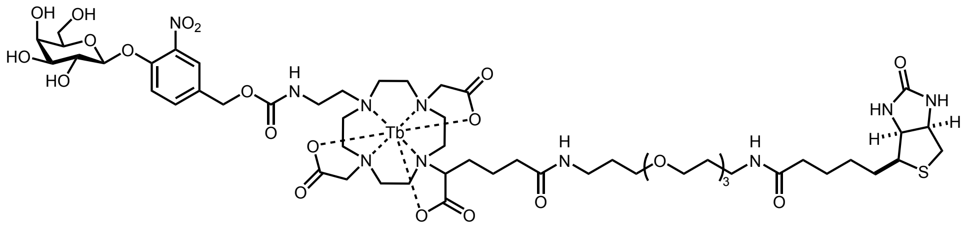

2,2'-(4-(1-carboxy-5,21-dioxo-25-(2-oxohexahydro-1*H*-thieno[3,4-*d*]imidazol-4-yl)-10,13,16-trioxa-6,20-diazapentacosyl)-10-(2-((((3-nitro-4-(((2*S*,3*R*,4*S*,5*R*,6*R*)-3,4,5-trihydroxy-6-(hydroxymethyl)tetrahydro-2*H*-pyran-2-yl)oxy)benzyl)oxy)carbonyl)amino)ethyl)-1,4,7,10-

tetraazacyclododecane-1,7-diyl)diacetic acid, terbium(III) (Tb1). The title compound was synthesized and purified in an analogous fashion to that reported for **Gd1**. LC/MS (ESI): Exact mass calcd. for $C_{54}H_{85}TbN_{10}O_{22}S$ $[M-H]^-$, 1415.47. Found 1415.34.

Relaxation measurements at 1.41 T. T_1 and T_2 values were acquired on a Bruker mq60 NMR analyzer equipped with Minispec V2.51 Rev.00/NT software (Billerica, MA, U.S.A.) at 1.41 T (60 MHz) and 37 °C. A 1 mM solution of either **Gd1** or **Gd2** at pH 7.4 was prepared in 100 mM MOPS buffer containing 1 mM $MgCl_2$ and 50 mM 2-mercaptoethanol. This sample was then serially diluted to yield five separate concentrations. Samples were incubated at 37 °C for 30 min and the T_1 and T_2 relaxation times measured. T_1 relaxation times were determined using an inversion recovery pulse sequence (t1_ir_mb) with the following parameters: four scans per point, 10 data points for fitting, mono-exponential curve fitting, phase cycling, 10 ms first pulse separation, and a recycle delay and final pulse separation ≥ 5T1. T_2 relaxation times were measured using a Carr–Purcell–Meiboom–Gill (CPMG) pulse sequence (t2_cp_mb) with the following parameters: four scans per point, mono-exponential curve fitting, phase cycling, 10 ms first pulse separation, 15 second recycle delay, 1 ms 90–180° pulse separation (tau), while altering the number of data points to ensure accurate mono-exponential curve fitting (500–10000 data points for fitting). Relaxivities were determined by taking the slope of a plot of $1/T_1$ (s^{-1}) or $1/T_2$ (s^{-1}) against the corresponding gadolinium concentration (mM) determined by ICP-MS.

UV/Vis Studies. UV-Vis studies were performed using a 96 well plate on either an Infinite M200 PRO or a BioTek Synergy 4 microplate reader. Absorbance readings at 420 nm were recorded in triplicate at 37 °C every five and 20 seconds on the Infinite M200 PRO and BioTek Synergy 4 microplate reader, respectively.

Luminescence lifetime measurements. The luminescence measurements were performed on a Varian eclipse spectrofluorimeter, equipped with a 450 W xenon arc lamp, a microsecond flash lamp and a red-sensitive photomultiplier (300–850 nm). The luminescence lifetime were recorded by excitation at 371 nm and emission at 545 nm. Emission was collected at a right angle to the excitation beam. The signal from 50 flashes were collected and averaged. Excitation slit width was set at 20 nm while the emission slit was at 10 nm. Total decay time of 35 ms was used with delay time of 0.05 ms and gate time of 0.2 ms. Luminescence decay curves were analyzed with Prism graphpad software. The experimental decay curves were fitted to a single exponential model using the Chi-squared criteria to discriminate the best exponential fit.

ICP-MS. Analysis of Gd(III) content in solutions and cell suspensions was performed as previously outlined.[6]

Complexation with avidin. The HABA/avidin reagent was reconstituted with 10 mL of water, and the assay performed in a manner similar to the instructions provided by the manufacturer. In a typical experiment, 10 µL aliquots of 80 µM sample in water were titrated into 450 µL of the reconstituted HABA/avidin solution. The decrease in absorbance at 500 nm (ΔA_{500}) due to the displacement of HABA from avidin was monitored using UV-visible spectroscopy. Equation **1** was used to calculate each ΔA_{500} plotted in the absorption titration curve. In this equation, V_0 is the initial volume of HABA/avidin solution, V_i is the total volume of sample solution added, A_{HABA} is the initial absorbance at 500 nm of the HABA/avidin solution, and $A_{HABA+sample}$ is the absorbance at 500 nm after addition of each aliquot to HABA/avidin. Absorption titration curves plotting ΔA_{500} vs [complex]:[avidin] were used to determine the equivalence point in each titration.

$$\Delta A_{500} = \left(\frac{V_0}{V_0 + V_i}\right) A_{HABA} - A_{HABA\,+\,sample} \quad \textbf{(1)}$$

Solution phantoms. Three separate solution phantoms were obtained for **Gd1** (0.15 mM, 100 mM MOPS, pH = 7.4, 37 °C) alone, complexed to avidin (20 U), and complexed to avidin in the presence of β-gal (2.6 mU) after 4 h. Following parameters were used for acquisition of images: repetition time (TR) = 1200 ms; echo time (TE) = 3.8 ms; slice thickness 10 mm; field of view (FOV) 75*100 mm, number of averages = 16, inversion time = 400 ms.

General Cell Culture. Dulbecco's modified phosphate buffered saline (DPBS), media, and dissociation reagents were purchased from Life Technologies (Carlsbad, CA, USA). CorningBrand® cell culture consumables (flasks, plates, etc.) and sera were purchased from VWR Scientific (Radnor, PA, USA). Rat 9L gliosarcoma cells stably overexpressing TfR and EGFR[7] were cultured in high glucose, phenol red containing Dublecco's modified eagle media (DMEM) supplemented with 10% fetal bovine serum (FBS) and 1% hygromycin. Prior to all experiments, cells were plated and allowed to incubate for 24 hours before dosing. Cells were harvested with 0.25% TrypLE for 10 min at 37 °C in a 5.0% CO_2 incubator. All doses were filtered with 0.2 μm sterile filters prior to administration. Cells were grown in a humidified incubator operating at 37 °C and 5.0% CO_2.

Cell Uptake. For time-dependent cell uptake, cells were plated at a density of 45,000 per well in 24-well plates. Cells were incubated with 20 μM Gd(III) targeted (6.6 μM Tf) and untargeted (0 μM Tf) contrast agent for 1, 2, 4, 8, or 24 hours. Following incubation, the media was aspirated, cells were washed with DPBS (2 x 500 μL), and detached with 100 μL TrypLE. Media was added to the cell suspensions (100 μL) and cells were centrifuged at 1000 x g for 5 min at 4 °C. The media was aspirated and replaced with 200 μL fresh media. A 50 μL aliquot was used for cell counting and 130 μL was used for quantification of Gd(III) by ICP-MS. For concentration-dependent uptake, the same procedure was followed with the following exception: cells were

incubated with concentrations of targeted and untargeted agent ranging from 0 – 45 μM Gd(III) for 2 hours.

Cell Counting and Viability After labeling experiments, cell count and viability were measured using a Guava EasyCyte Mini Personal Cell Analyzer (EMD Millipore, Billerica, MA). Briefly, a 50 μL aliquot of cell suspension was mixed with Guava ViaCount reagent to reach a total volume of 200 μL. The viability and cell count were determined using ViaCount module software.

References

1. Pangborn, A. B.; Giardello, M. A.; Grubbs, R. H.; Rosen, R. K.; Timmers, F. J. *Organometallics* **1996**, *15*, 1518-1520.
2. Armarego, W. L. F.; Chai, C. L. L. *Purification of Laboratory Chemicals*; Butterworth-Heinemann, 2003.
3. Giardiello, M.; Lowe, M. P.; Botta, M. *Chem. Commun.* **2007**, 4044-4046.
4. Szalecki, W. *Bioconjugate Chemistry* **1996**, *7*, 271-273.
5. Renaud de la Faverie, A.; Hamon, F.; Di Primo, C.; Largy, E.; Dausse, E.; Delaurière, L.; Landras-Guetta, C.; Toulmé, J.-J.; Teulade-Fichou, M.-P.; Mergny, J.-L. *Biochimie* **2011**, *93*, 1357-1367.
6. Harrison, V. S. R.; Carney, C. E.; Macrenaris, K. W.; Meade, T. J. *Chem. Commun.* **2014**, *50*, 11469-11471.
7. Broome, A.-M.; Bhavsar, N.; Ramamurthy, G.; Newton, G.; Basilion, J. P. *Molecular Pharmaceutics* **2010**, *7*, 60-74.

Figure S1. ^{1}H NMR spectrum of **4** (500 MHz, $CDCl_3$)

Figure S2. ^{13}C NMR spectrum of **4** (126 MHz, $CDCl_3$)

Figure S3. ^{1}H NMR spectrum of **5** (500 MHz, $CDCl_3$)

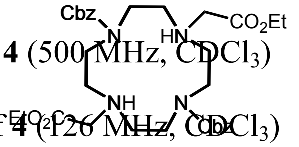

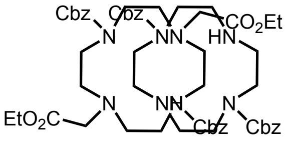

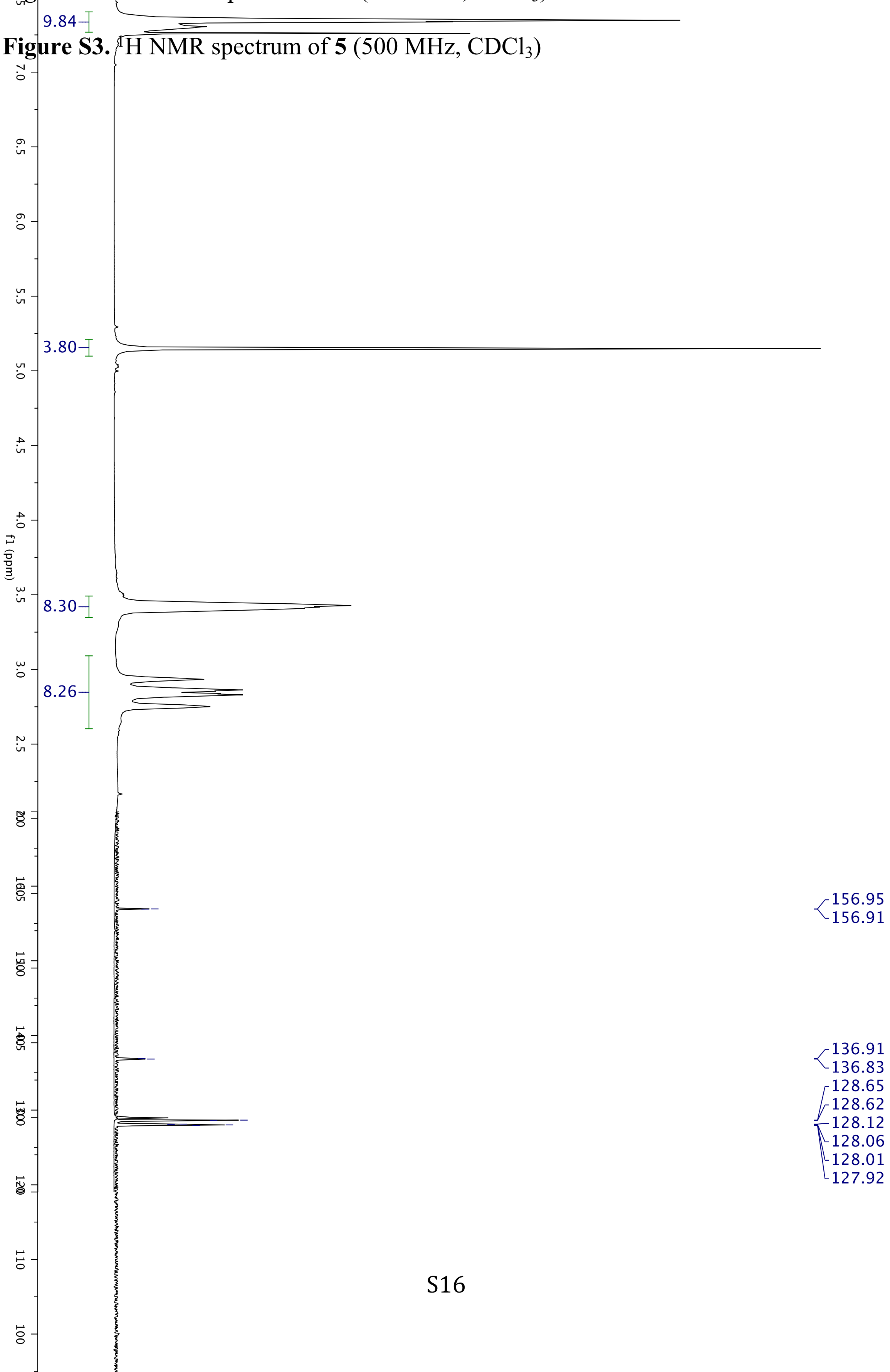

Figure S4. ^{13}C NMR spectrum of **5** (126 MHz, $CDCl_3$)

Figure S5. ^{1}H NMR spectrum of **6** (500 MHz, $CDCl_3$)

Figure S6. ^{13}C NMR spectrum of **6** (126 MHz, $CDCl_3$)

Figure S7. ^{1}H NMR spectrum of **7** (500 MHz, $CDCl_3$)

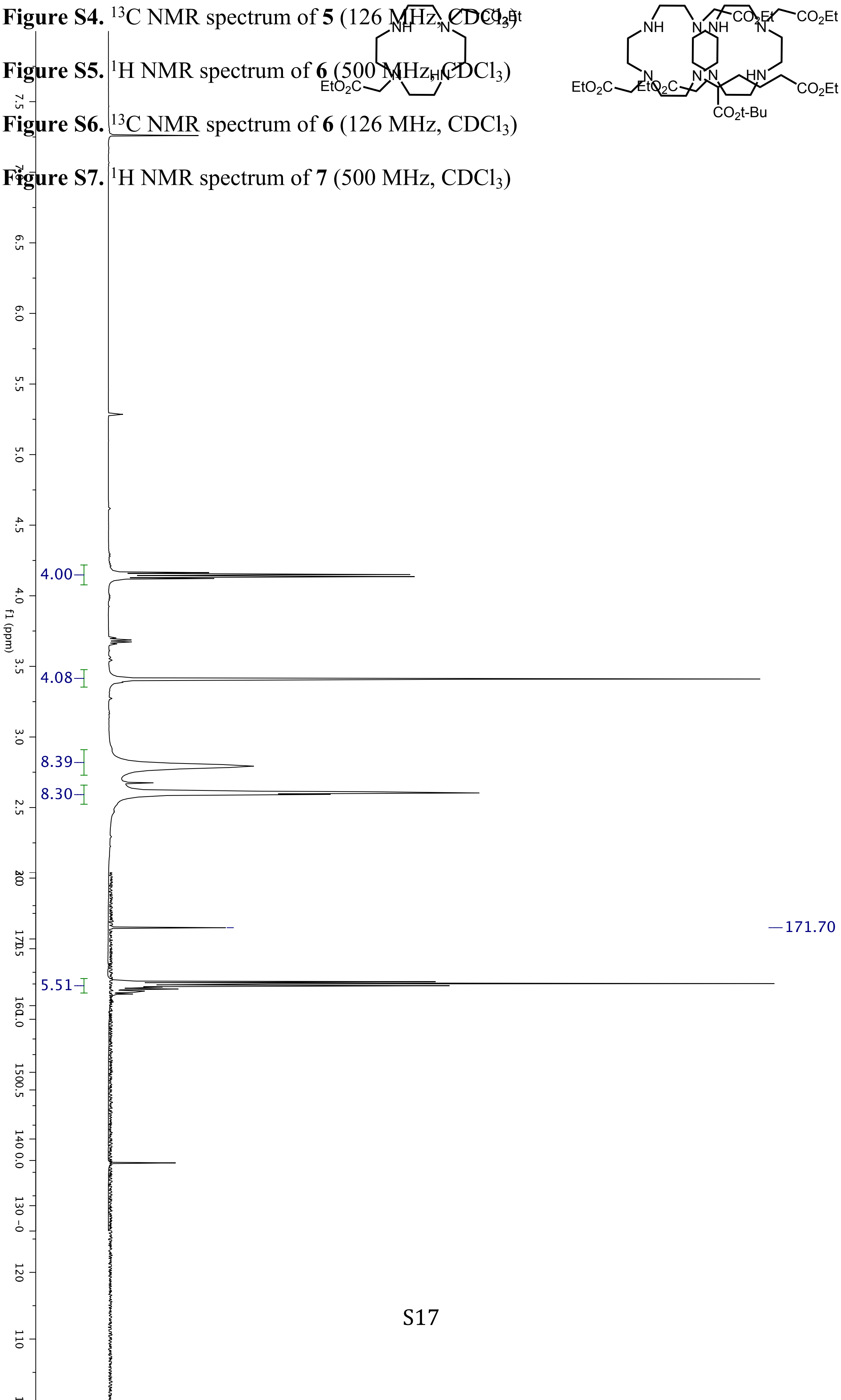

Figure S8. ^{13}C NMR spectrum of **7** (126 MHz, $CDCl_3$)

Figure S9. ^{1}H NMR spectrum of **8** (500 MHz, $CDCl_3$)

Figure S10. ^{13}C NMR spectrum of **8** (126 MHz, $CDCl_3$)

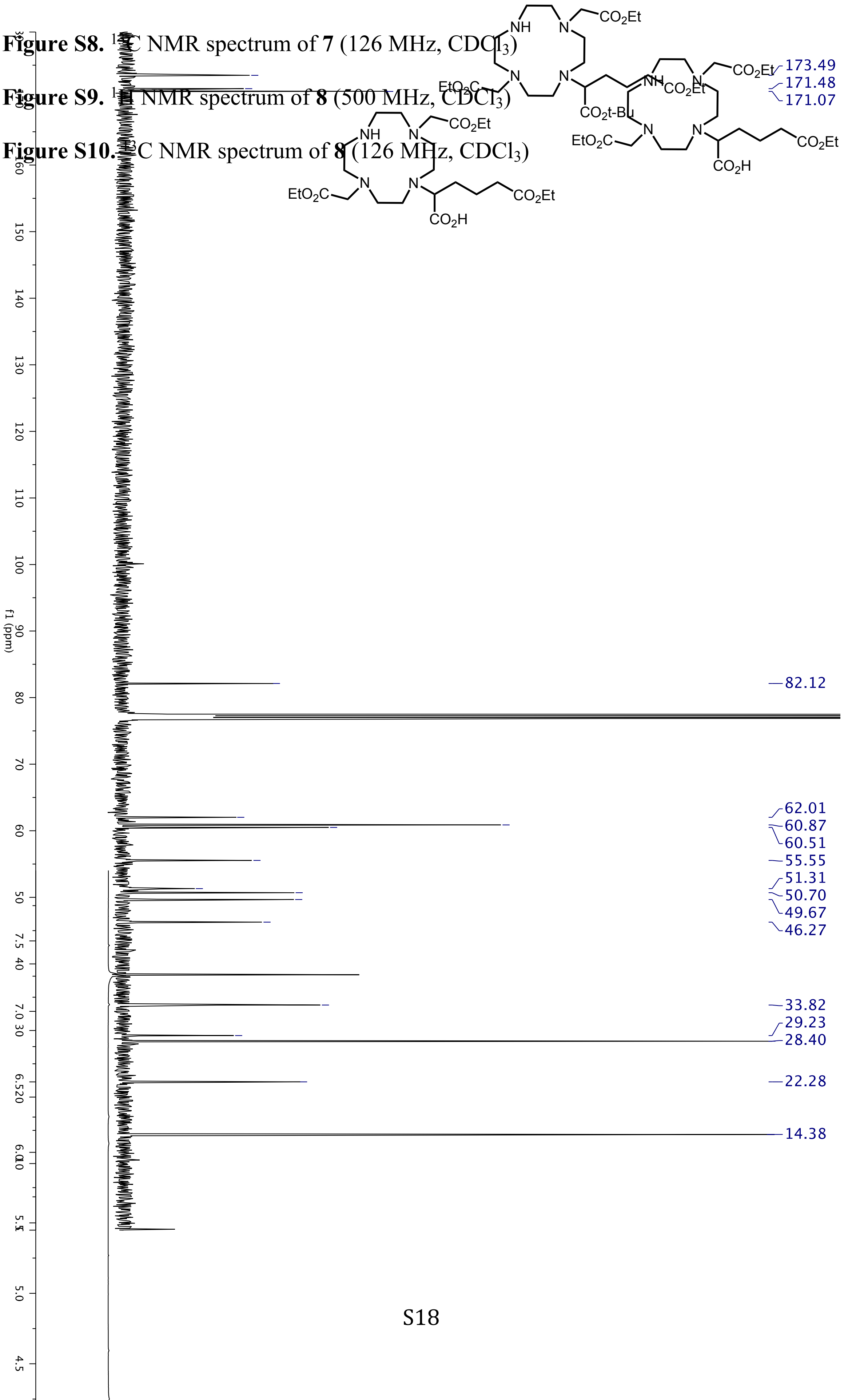

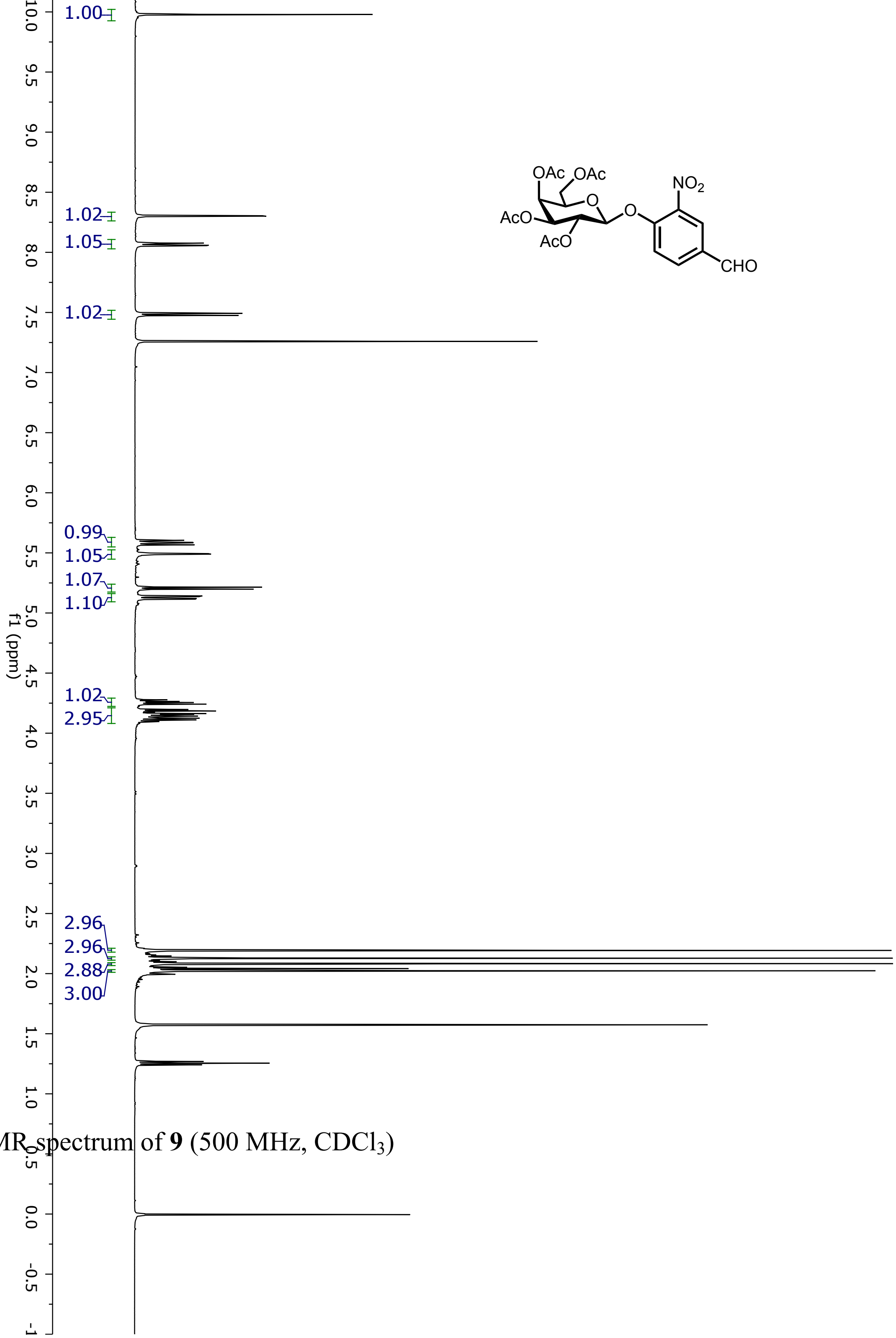

Figure S11. ^{1}H NMR spectrum of **9** (500 MHz, $CDCl_3$)

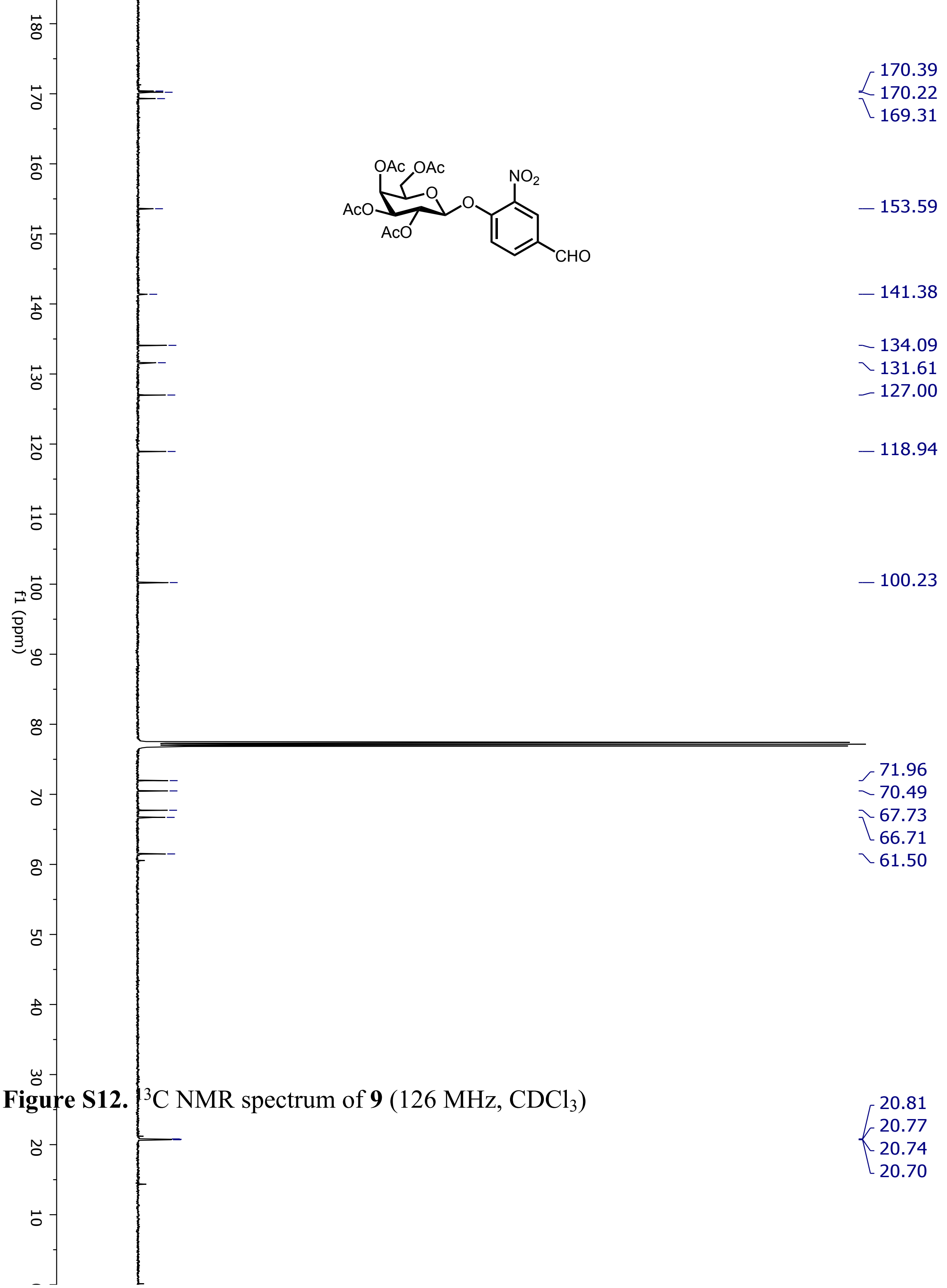

Figure S12. ^{13}C NMR spectrum of **9** (126 MHz, $CDCl_3$)

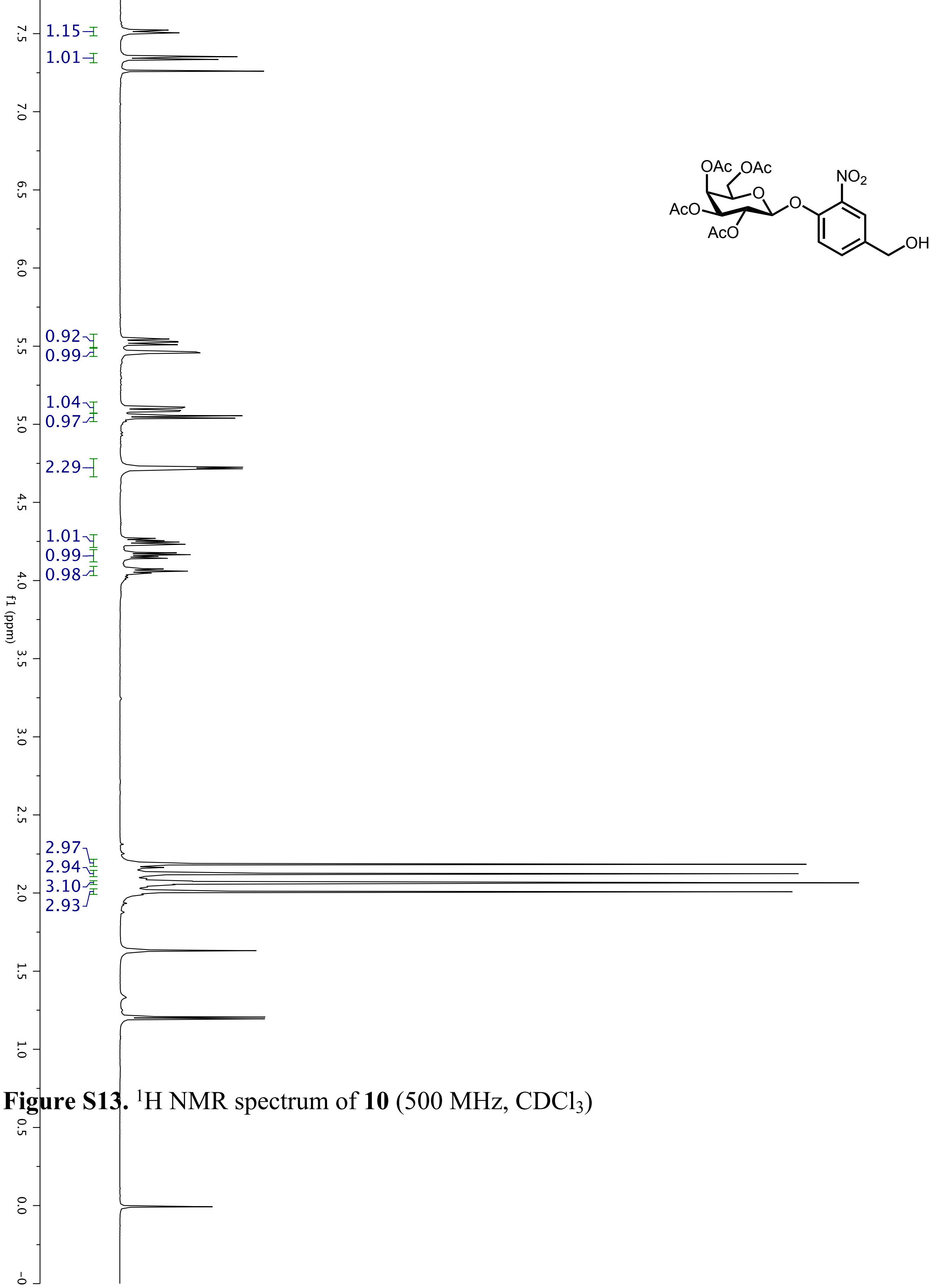

Figure S13. ^{1}H NMR spectrum of **10** (500 MHz, $CDCl_3$)

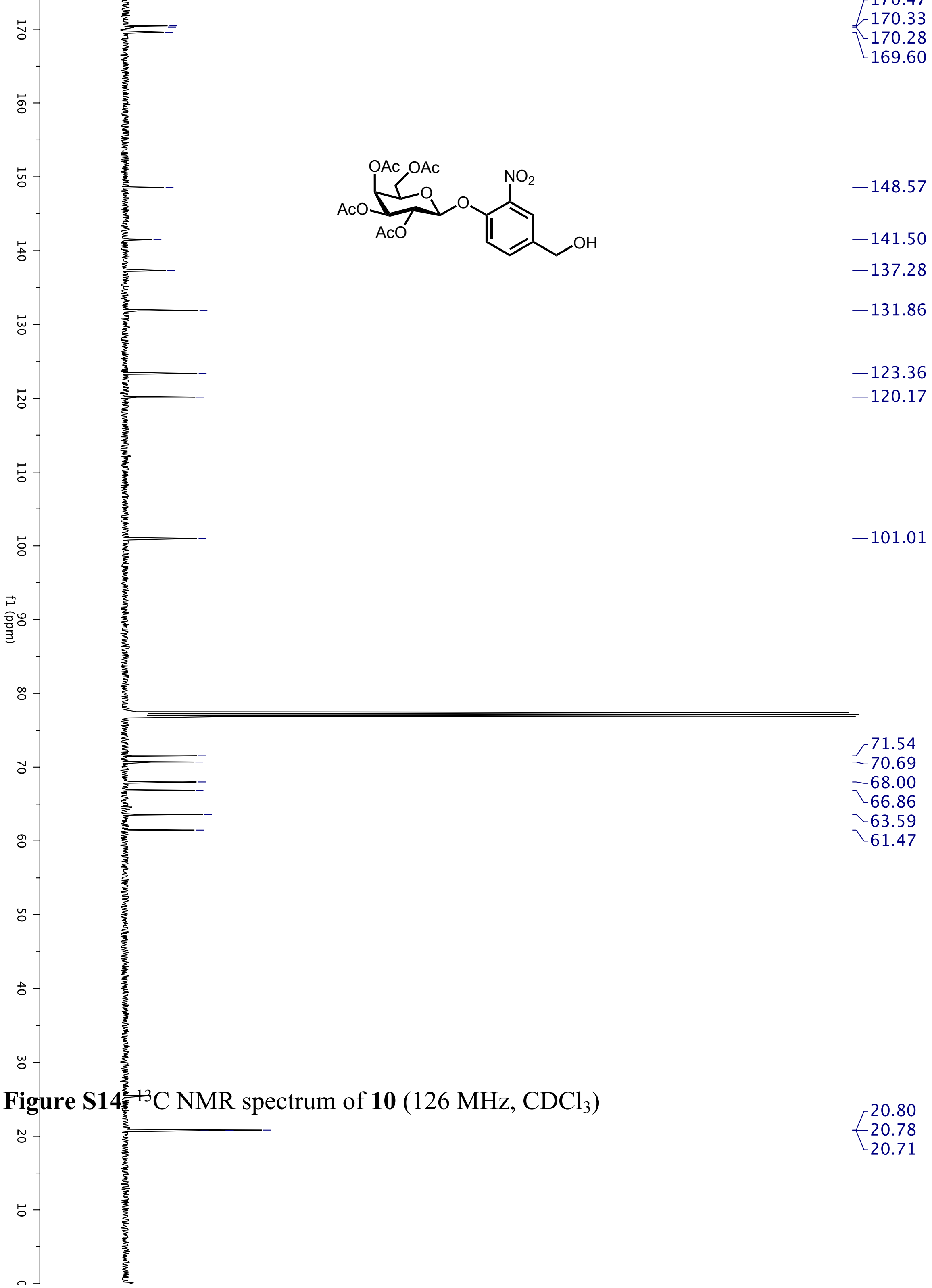

Figure S14. ^{13}C NMR spectrum of **10** (126 MHz, $CDCl_3$)

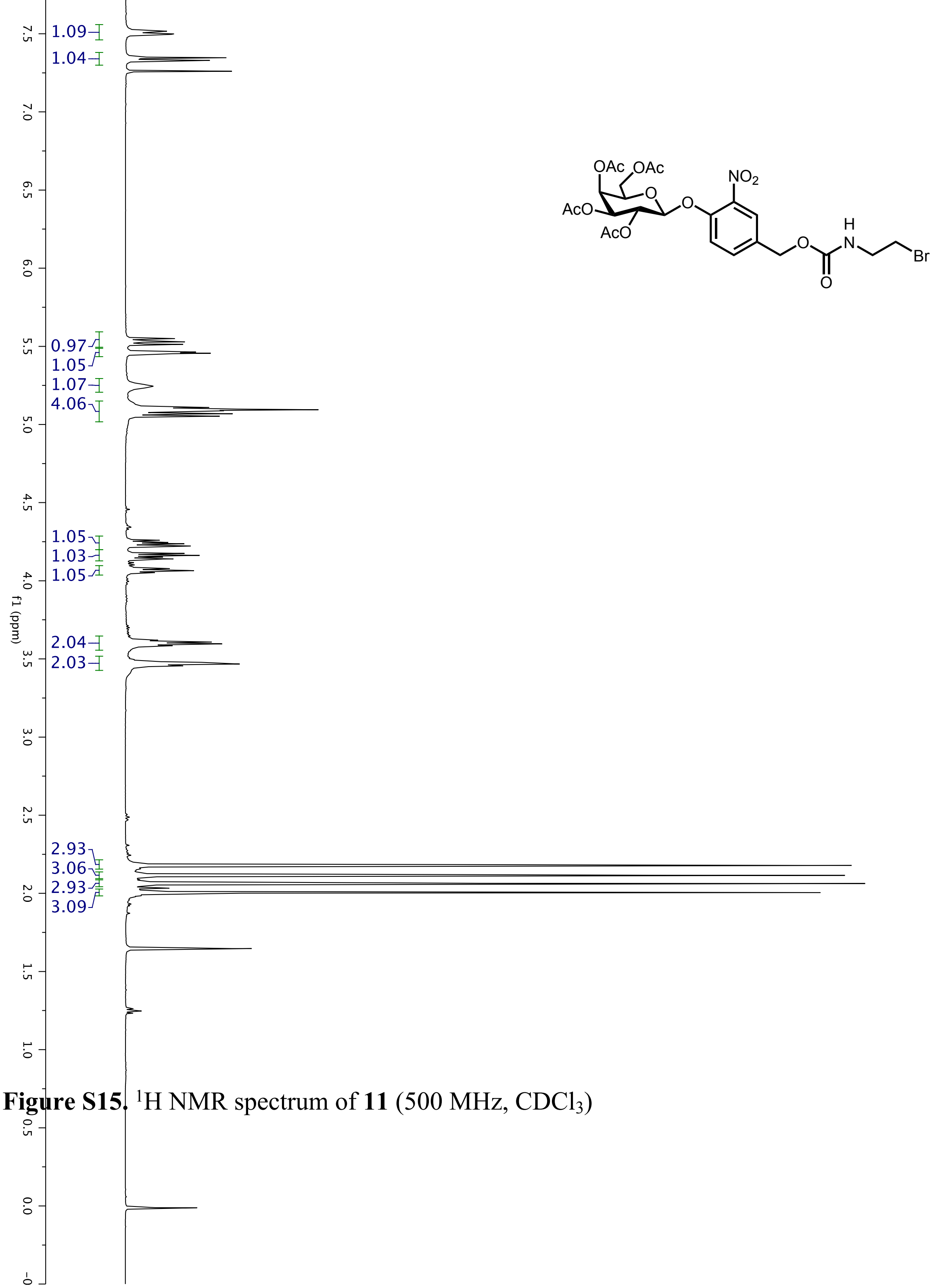

Figure S15. ^{1}H NMR spectrum of **11** (500 MHz, $CDCl_3$)

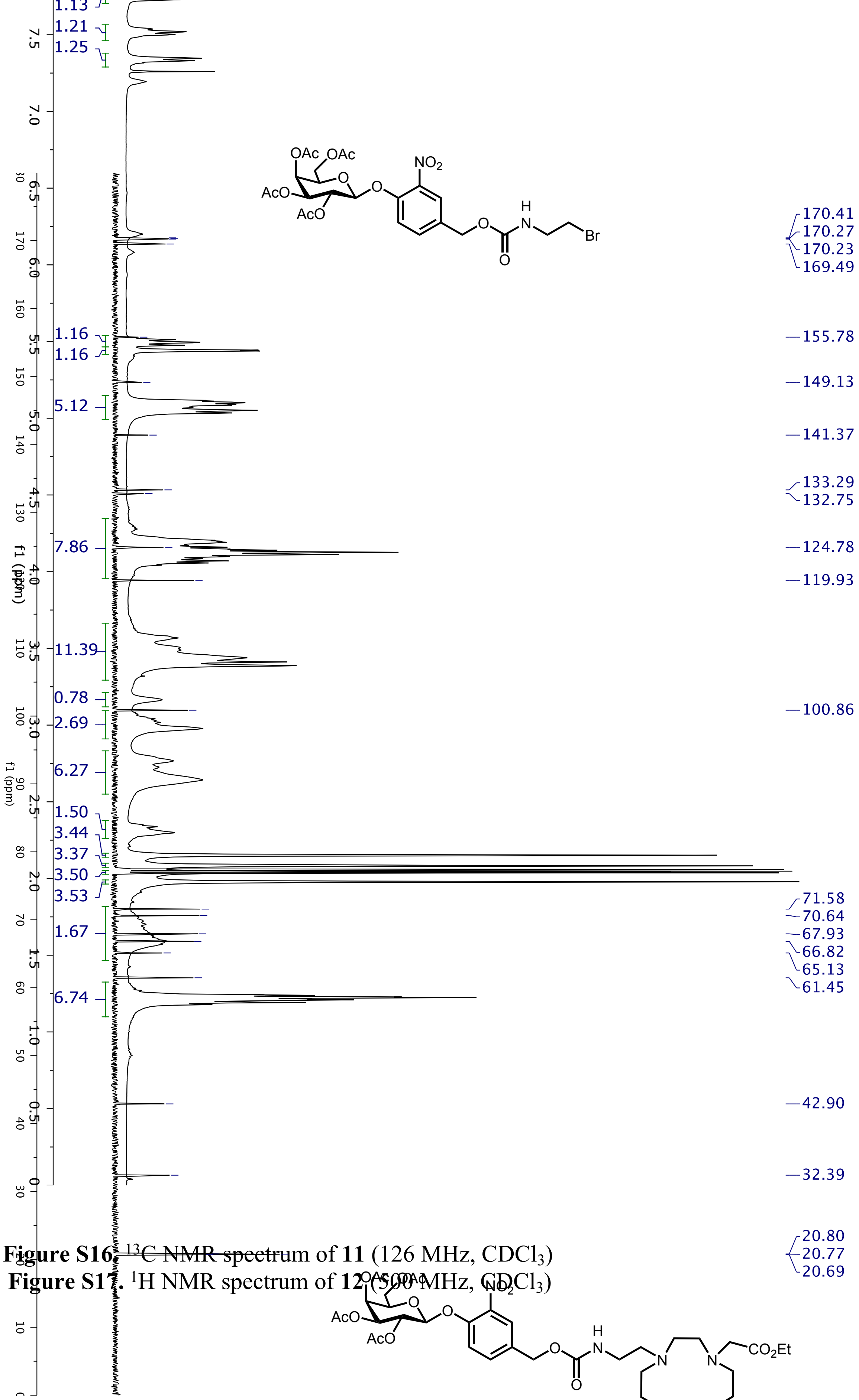

Figure S16. ^{13}C NMR spectrum of **11** (126 MHz, $CDCl_3$)

Figure S17. ^{1}H NMR spectrum of **12** (500 MHz, $CDCl_3$)

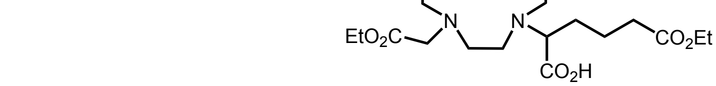

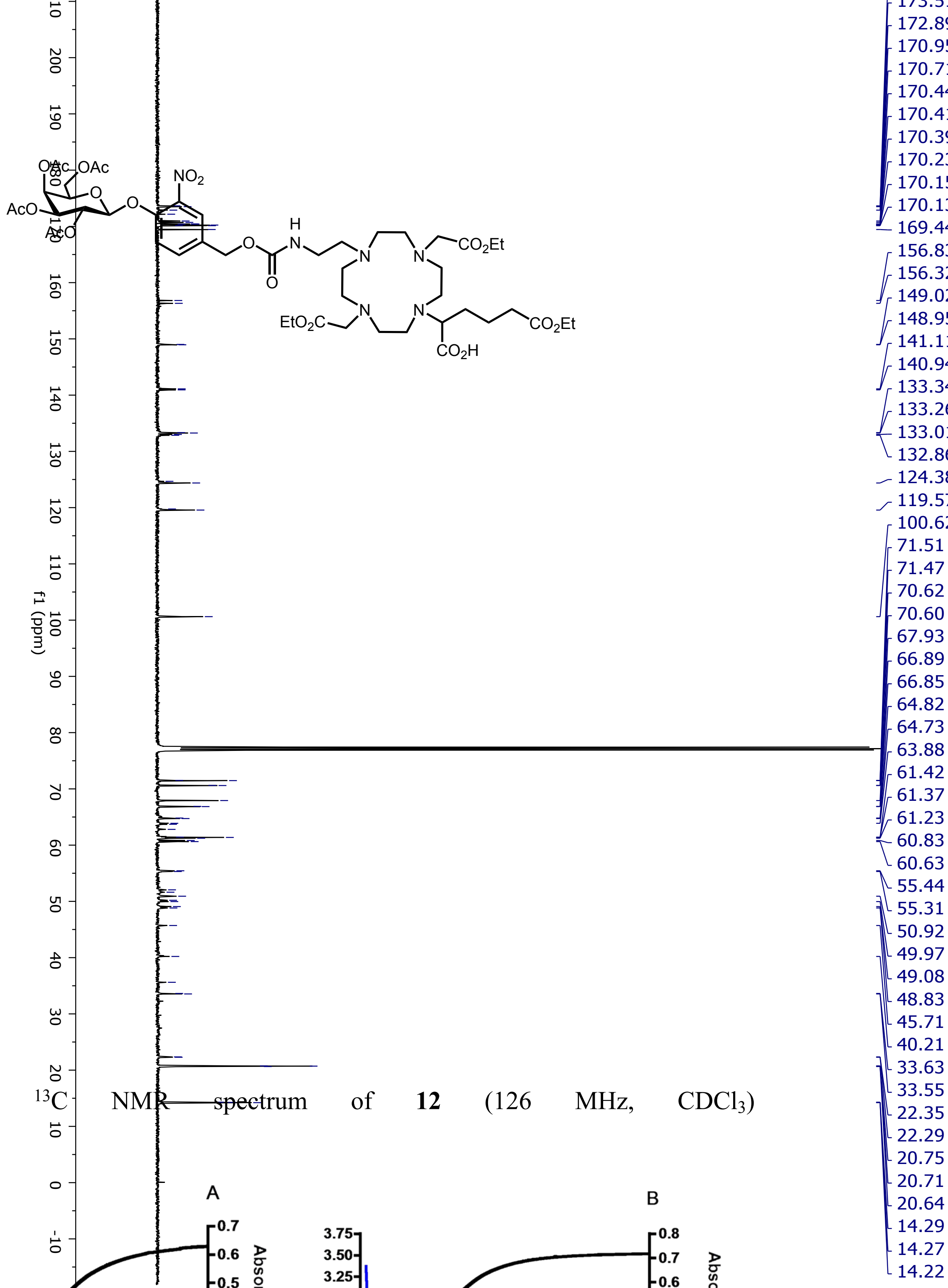

Figure S18. ^{13}C NMR spectrum of **12** (126 MHz, $CDCl_3$)

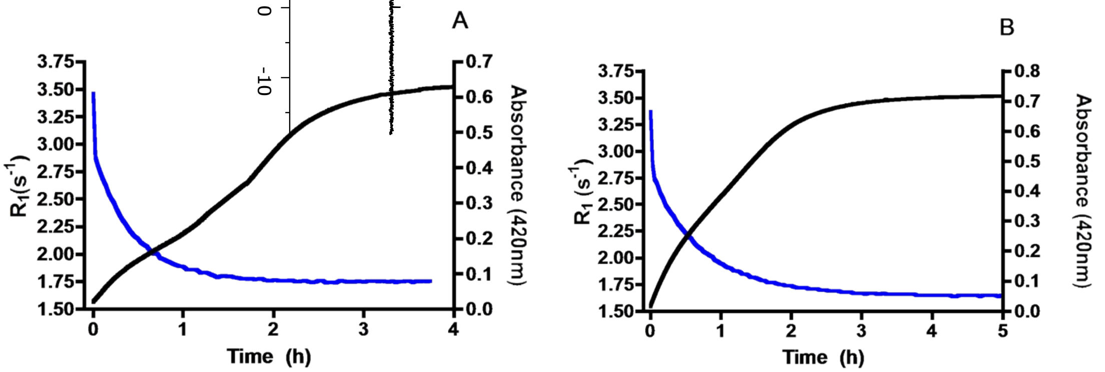

Figure S19. Kinetics of enzyme activation as measured by a change in the longitudinal relaxation

rate (blue trace) and absorbance (black trace) for (A) **Gd2** (0.35 mM, 100 mM MOPS, pH 7.4, 2.4 μU/μL) and (B) **Gd1** (0.32 mM, 100 mM MOPS, pH 7.4, 2.7 μU/μL).

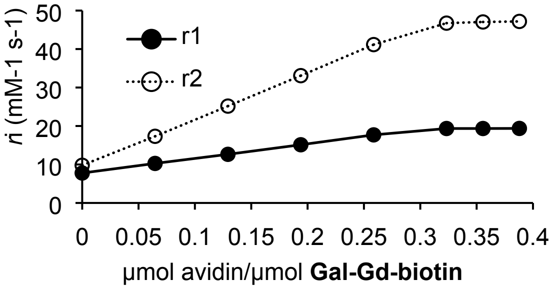

Figure S20. Relaxivity values (r_1 and r_2) as a function of avidin equivalents for **Gd2** (0.17 mM).

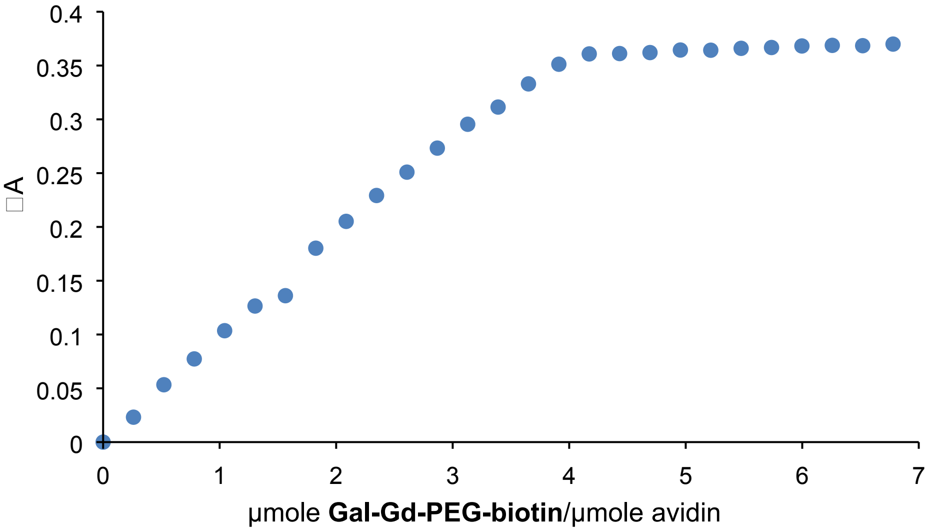

Figure S21. Absorption titration curve of HABA/avidin assay with **Gd1**.

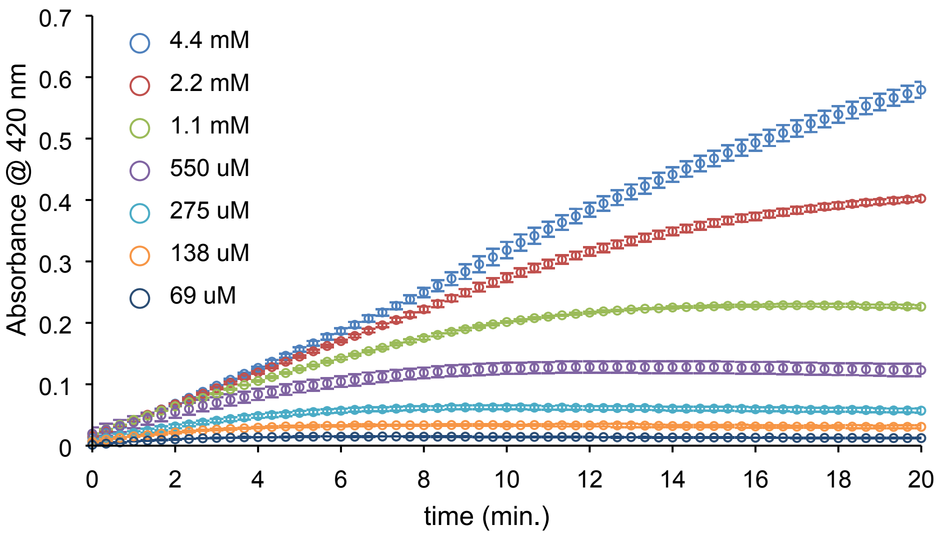

Figure S22. Spectrophotometric response of ONPG in the presence of 1 μM biotinylated β-gal.

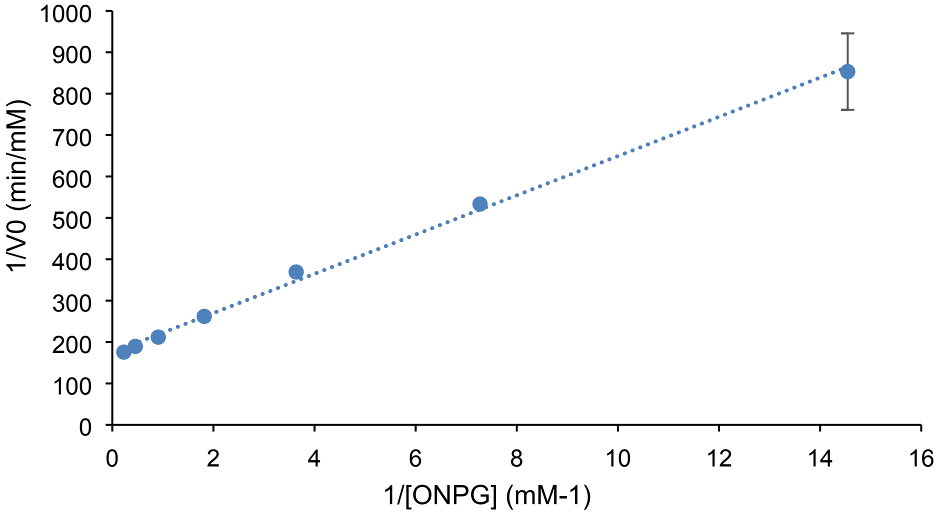

Figure S23. Lineweaver-Burk plot for measurements presented in **Figure S22**. $K_m = 0.270$ mM; $k_{cat} = 0.095\ s^{-1}$

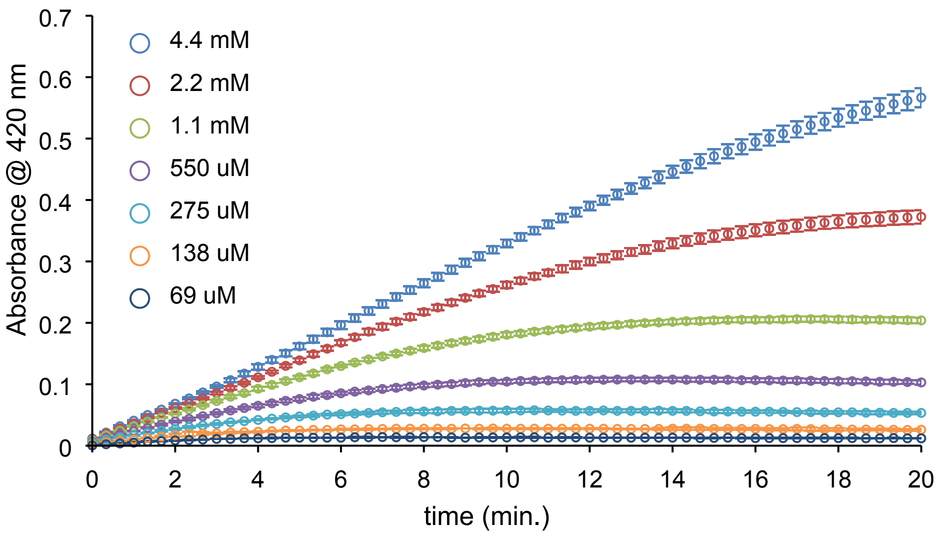

Figure S24. Spectrophotometric response of ONPG in the presence of 0.25 µM 4:1 biotinylated β-gal:avidin.

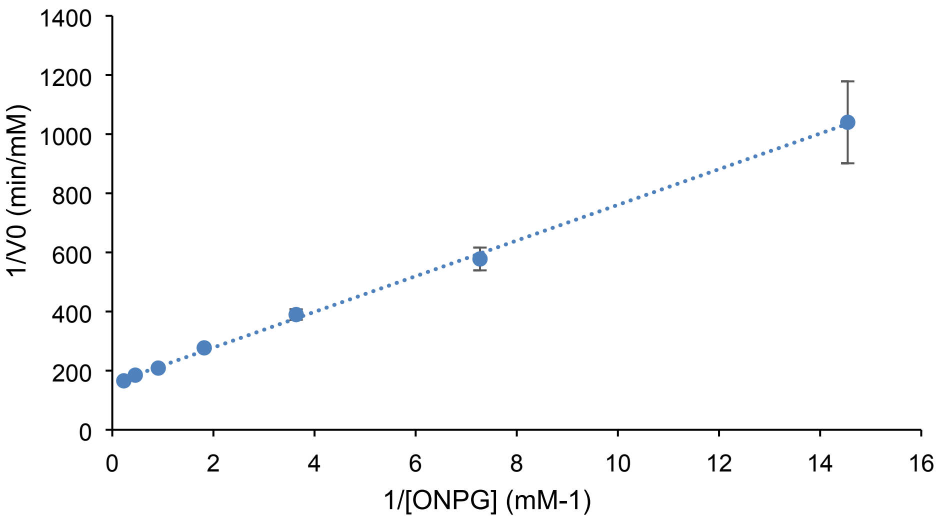

Figure S25. Lineweaver-Burk plot for measurements presented in **Figure S24**. K_m = 0.383 mM; k_{cat} = 0.106 s^{-1}

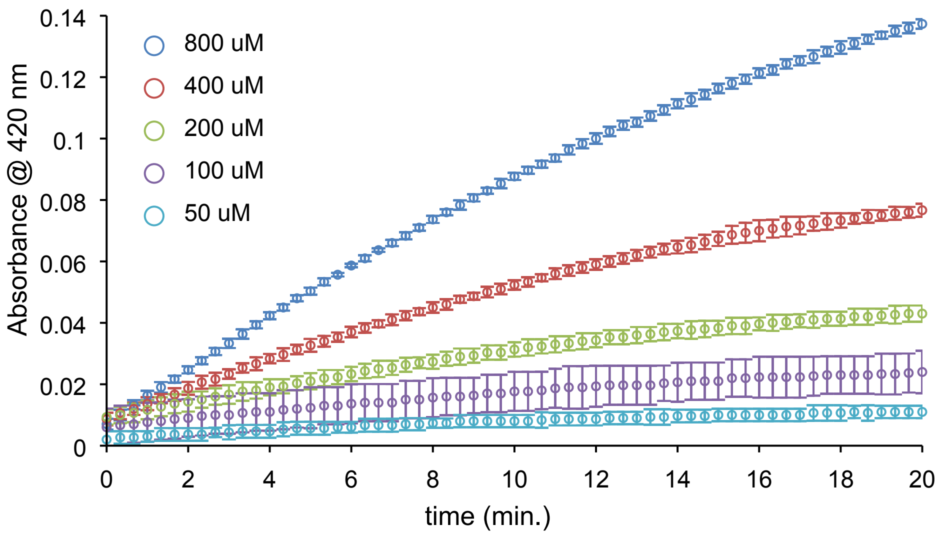

Figure S26. Spectrophotometric response of **Gd1** in the presence of 1 μM biotinylated β-gal.

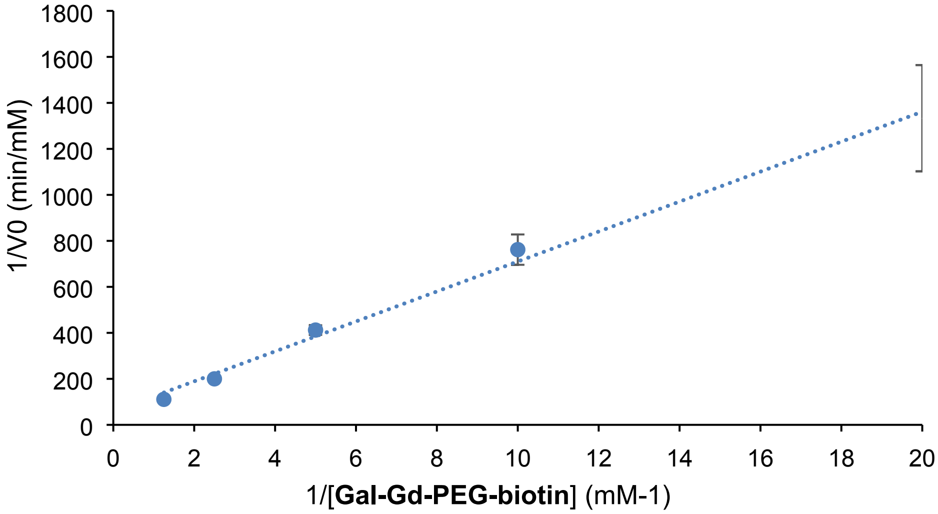

Figure S27. Lineweaver-Burk plot for measurements presented in **Figure S26**. K_m = 1.11 mM; k_{cat} = 0.283 s^{-1}.

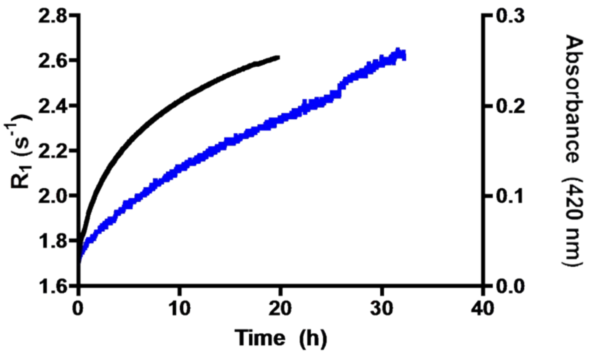

Figure S28. Kinetics of enzyme activation of **Gd1** (0.13 mM, 100 mM MOPS, pH 7.4, 44 µU/µL) as measured by a change in the longitudinal relaxation rate (blue trace) and absorbance (black trace).

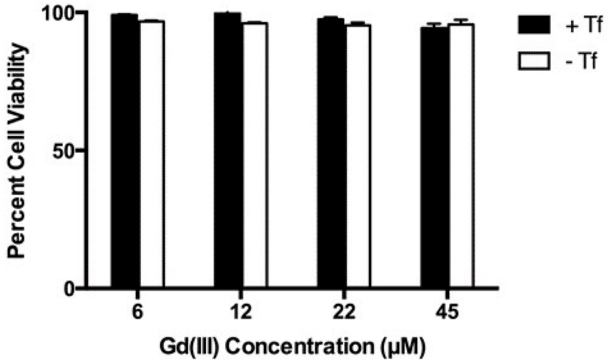

Figure S29. Cells incubated with various concentrations of targeted and untargeted contrast agent show viability greater than 90%.
